# Graded and threshold effects of body mass index on perioperative outcomes after pancreaticoduodenectomy: a meta-analysis incorporating an institutional cohort

**DOI:** 10.3389/fsurg.2026.1846295

**Published:** 2026-08-21

**Authors:** Yingyue Pan, Yi Chen, Yanyu Qiu, Ke Zhang, Xiaodong He, Naishi Li, Xianlin Han

**Affiliations:** 1 Department of Endocrinology, Key Laboratory of Endocrinology of National Health Commission, Peking Union Medical College Hospital, Chinese Academy of Medical Sciences and Peking Union Medical College, Beijing, China; 2 Department of General Surgery, Peking Union Medical College Hospital, Chinese Academy of Medical Sciences and Peking Union Medical College, Beijing, China; 3 State Key Laboratory of Complex Severe and Rare Diseases, Peking Union Medical College Hospital, Chinese Academy of Medical Sciences and Peking Union Medical College, Beijing, China; 4 Department of Medical Records, Peking Union Medical College Hospital, Chinese Academy of Medical Sciences and Peking Union Medical College, Beijing, China; 5 WHO Family of International Classifications Collaborating Center of China, Peking Union Medical College Hospital, Beijing, China

**Keywords:** body mass index, meta-analysis, pancreatic fistula, pancreaticoduodenectomy, postoperative complications

## Abstract

**Background:**

The influence of body mass index (BMI) on perioperative results after pancreaticoduodenectomy (PD) remains controversial. This study aimed to clarify how different BMI strata influence operative complexity, postoperative morbidity, and mortality.

**Methods:**

We performed a meta-analysis and systematic review of 25 studies, including one institutional cohort, evaluating BMI-related perioperative and survival outcomes in patients undergoing PD. The outcomes assessed included operative time, estimated blood loss, postoperative complications, clinically relevant pancreatic fistula, reoperation, perioperative mortality, and overall survival.

**Results:**

BMI ≥25 kg/m^2^ was linked to prolonged operative time, higher blood loss, and a greater risk of postoperative complications, including clinically relevant pancreatic fistula, delayed gastric emptying, and major complications, without a significant increase in perioperative mortality. In contrast, BMI ≥30 kg/m^2^ was associated with an increased risk of perioperative mortality. Analyses across BMI categories demonstrated a progressive increase in operative complexity with increasing BMI, whereas perioperative mortality exhibited a threshold pattern, with a marked increase observed primarily at BMI ≥30 kg/m^2^. No notable relationship was found between BMI and long-term overall survival.

**Conclusions:**

Overweight (BMI ≥25 kg/m^2^) is primarily associated with increased operative complexity and moderate morbidity, whereas obesity (BMI ≥30 kg/m^2^) represents a threshold associated with perioperative mortality. These findings support BMI-based risk stratification and may inform individualized perioperative management in patients undergoing PD.

## 1 Introduction

Pancreaticoduodenectomy (PD) remains a primary curative treatment for periampullary and pancreatic malignancies and is among the most technically complex procedures in abdominal surgery (1). Although operative mortality has decreased to below 3% in high-volume centers (2–4), postoperative morbidity remains substantial, with pooled rates of overall complications approaching 55% (5). Major complications, including postoperative pancreatic fistula (POPF), delayed gastric emptying (DGE), hemorrhage, and sepsis, notably impact recovery, extend hospitalization, and contribute to increased healthcare costs (6, 7). Therefore, accurate preoperative risk stratification is essential to optimize patient selection and perioperative management (8).

Obesity has emerged as a global epidemic and is increasingly encountered among surgical candidates (9, 10). Defined by body mass index (BMI), obesity is associated with multiple comorbidities and adverse surgical outcomes (11–13). Excess visceral fat may impair operative exposure, prolong operative time, increase blood loss, and compromise reconstruction quality (14–16). Beyond these mechanical factors, accumulating evidence suggests that obesity-induced alterations in tissue biology play a critical role in adverse surgical outcomes. In particular, visceral fat accumulation is linked to chronic low-grade inflammation, marked by heightened infiltration of adipose tissue macrophages and the release of pro-inflammatory cytokines, which may impair anastomotic healing and increase susceptibility to infectious complications (17, 18). Moreover, pancreatic fat infiltration has been recognized as a significant risk factor for POPF following PD (19–22), providing a plausible mechanistic link between elevated BMI and postoperative complications.

However, the impact of BMI on outcomes after PD remains inconsistent across the literature. Although several studies have shown that a higher BMI is linked to greater operative complexity and postoperative complications (11, 13, 16, 23), especially POPF (9, 21), others have found no significant differences in overall morbidity (24) or mortality (25). The association between BMI and long-term survival has been less consistently reported and remains inconclusive. Reported findings include worse survival (26–28), no difference (19, 29, 30), or a potential “obesity paradox” (31, 32). Although several recent meta-analyses have addressed this topic, they have either pooled heterogeneous pancreatic resection types or relied primarily on dichotomous BMI comparisons, leaving the distinction between graded and threshold effects unexplored (33, 34).

Several methodological limitations may contribute to these conflicting findings. First, substantial variability exists in BMI classification, with most studies adopting dichotomous thresholds (e.g., ≥25 or ≥30 kg/m^2^), which may not fully capture the relationship between BMI and surgical risk (23, 35). Second, heterogeneity in study populations, surgical techniques, and outcome definitions further complicates comparisons across studies. Importantly, it remains unclear whether perioperative risk increases at lower BMI thresholds (≥25 kg/m^2^) or becomes clinically significant only in patients with obesity (≥30 kg/m^2^), which directly affects risk stratification and surgical decision-making.

Thus, a thorough and stratified quantitative analysis is needed to clarify the clinical impact of BMI in patients undergoing PD. In this systematic review and meta-analysis, we assessed the relationship between BMI and perioperative as well as long-term outcomes using both dichotomous comparisons (BMI ≥25 vs. <25 kg/m^2^ and BMI ≥30 vs. <30 kg/m^2^) and three-category stratification (<25, 25–30, and ≥30 kg/m^2^).

The primary outcomes included clinically significant POPF and major complications, while secondary outcomes included overall complications, operative parameters, perioperative mortality, and overall survival. Importantly, this study was designed to address three key questions: whether increasing BMI is associated with progressively greater operative complexity, whether a distinct risk threshold exists at BMI ≥30 kg/m^2^ for severe adverse outcomes, and whether BMI-related risk follows a graded or threshold-dependent pattern. By addressing these questions, we aim to provide evidence to refine risk stratification and inform perioperative management in patients undergoing PD.

## 2 Materials and methods

### 2.1 Study design and eligibility criteria

This systematic review and meta-analysis assessed the effect of body mass index (BMI) on perioperative outcomes after PD, following the guidelines set by the Preferred Reporting Items for Systematic Reviews and Meta-Analyses (PRISMA 2020). The study protocol was registered prospectively in PROSPERO under the ID (CRD420261282972).

Eligible studies were cohort studies reporting perioperative or survival outcomes in adult patients undergoing PD, stratified by BMI (≥25 or ≥30 kg/m^2^). Exclusion criteria included reviews, case reports, editorials, non-human studies, conference abstracts, overlapping cohorts, and studies lacking extractable outcome data.

Two reviewers independently conducted the study selection and data extraction, with any disagreements resolved through discussion or by consulting a third reviewer. Extracted data included patient demographics, study characteristics, BMI definitions, and perioperative outcomes.

In addition, an institutional retrospective cohort of 120 patients who underwent PD at Peking Union Medical College Hospital (PUMCH) between 2017 and 2023 was included. Data collection and outcome definitions aligned with those used in the included studies.

### 2.2 Search strategy

A systematic literature search was conducted in PubMed, Embase, and the Cochrane Library from database inception to March 13, 2026. The search strategy combined controlled vocabulary (MeSH in PubMed and Cochrane Library, and Emtree terms in Embase) with free-text terms.

Search terms related to BMI included “obesity,” “overweight,” “body mass index,” and related synonyms (e.g., adiposity, BMI, elevated BMI). Terms related to pancreatic surgery included “pancreaticoduodenectomy,” “pancreatoduodenectomy,” “Whipple procedure,” “pancreatectomy,” and related expressions (e.g., pancreatic resection, pancreatic surgery). Boolean operators (AND/OR) were employed to combine terms, with adaptations specific to each database.

The reference lists of relevant articles were manually reviewed to identify additional studies. All records were imported into EndNote (version 21) for duplicate removal. The complete search strategies for each database are detailed in Supplementary Table S1.

### 2.3 Exposure definition and outcomes

BMI was calculated by dividing weight (kg) by the square of height (m^2^). Two analytical approaches were applied: (1) dichotomous comparisons (BMI ≥25 vs. <25 kg/m^2^; BMI ≥30 vs. <30 kg/m^2^), and (2) categorical analyses stratifying patients into three groups (<25, 25–30, and ≥30 kg/m^2^) to assess graded associations.

The BMI thresholds of 25 and 30 kg/m^2^ were chosen according to the World Health Organization (WHO) classification for overweight and obesity. These thresholds are widely adopted in clinical research and provide a standardized framework for risk stratification. Applying both thresholds allowed us to distinguish between incremental (overweight) and potential threshold (obesity) effects of BMI on perioperative outcomes.

Primary outcomes included overall postoperative complications, major complications (Clavien–Dindo grade ≥ III), clinically relevant POPF (grade B/C), delayed gastric emptying, postoperative hemorrhage, and perioperative mortality (defined as in-hospital or 30-day mortality). Secondary outcomes included operative time, estimated blood loss, reoperation rate, and overall survival when reported.

### 2.4 Quality assessment and statistical analysis

Study quality was evaluated using the Newcastle–Ottawa Scale (NOS), with scores of 7 or higher deemed high quality. Two reviewers independently conducted the quality assessment. The detailed NOS scores are presented in Supplementary Table S2, and the quality summary and subgroup analysis are shown in Supplementary Figure S1.

Meta-analyses were performed using Review Manager (RevMan, version 5.4) and R software. Dichotomous outcomes were expressed as odds ratios (ORs), and continuous outcomes as mean differences (MDs), both with 95% confidence intervals (CIs). Heterogeneity was evaluated using the *I*^2^ statistic and Cochran's *Q* test; a random-effects model was applied when *I*^2^ > 50%, and a fixed-effects model was used otherwise.

To explore patterns of association across BMI categories, pooled effect estimates from pairwise comparisons were examined descriptively to assess potential graded or threshold effects.

Meta-regression analyses were performed to explore potential sources of heterogeneity, including publication year, sample size, mean age, male proportion, and geographic region. Exploratory subgroup analyses were performed within each BMI comparison for clinically relevant POPF according to outcome definition, surgical approach, geographic region, and publication year, with formal tests for subgroup differences. Surgical-approach subgroup analyses were also conducted for the primary outcomes. Sensitivity analyses included a leave-one-out approach and exclusion of the PUMCH cohort from every pooled analysis to which it contributed. Publication bias was evaluated using funnel plots and Egger's test for outcomes with sufficient studies. A *P* value of less than 0.05 (two-tailed) was regarded as statistically significant.

## 3 Results

### 3.1 Study selection and characteristics

**Figure 1:**
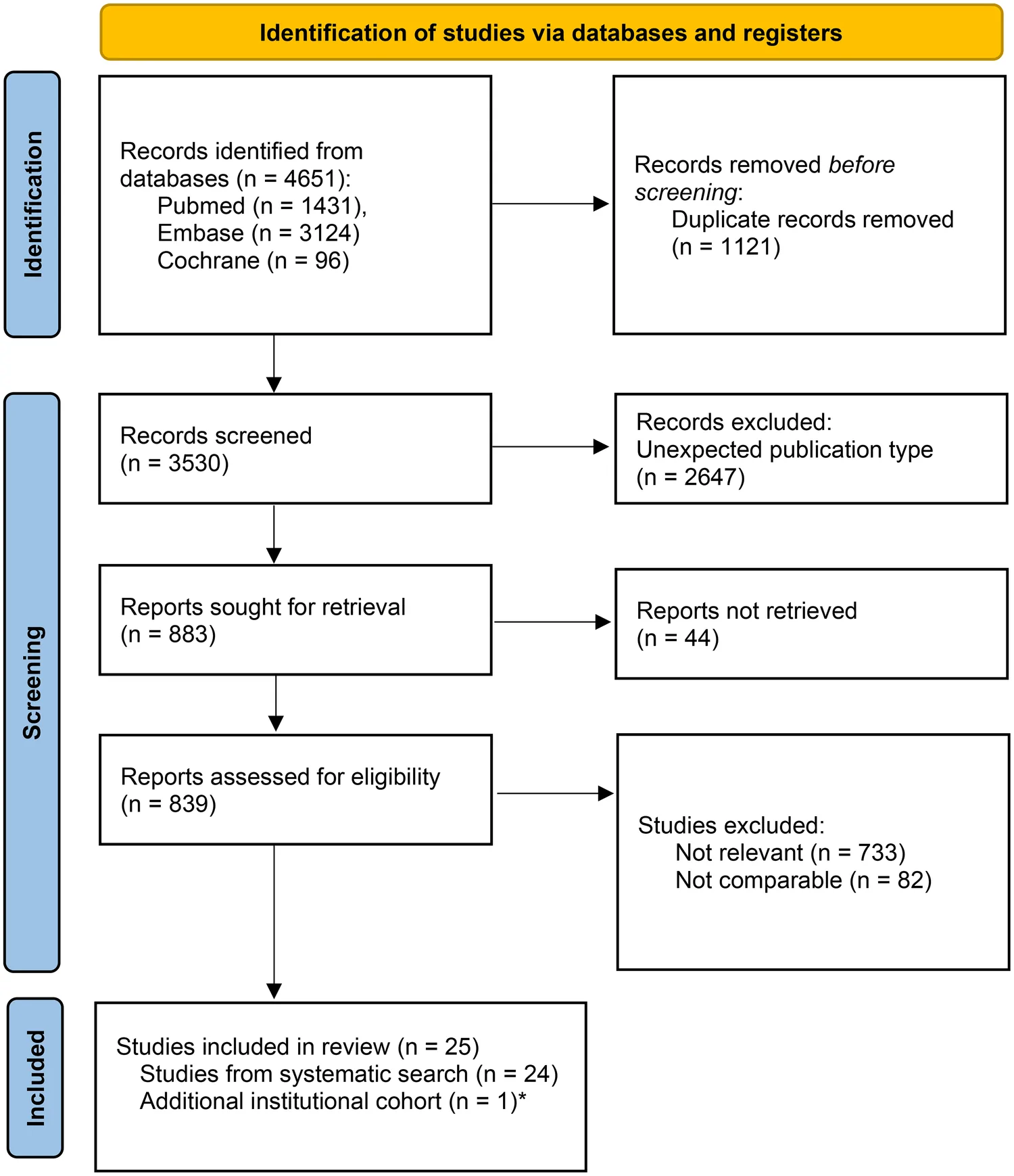
PRISMA flow diagram of study selection. Flow diagram illustrating the process of study identification, screening, eligibility assessment, and inclusion in accordance with PRISMA guidelines. *An additional institutional retrospective cohort from Peking Union Medical College Hospital (2017–2023; *n* = 120) was included in the final analysis, resulting in a total of 25 studies.

A comprehensive literature search identified 4,651 records from PubMed, Embase, and the Cochrane Library. After eliminating duplicates and screening according to predefined eligibility criteria, 25 studies were included in the final quantitative synthesis, consisting of 24 published studies and one institutional cohort. The clinicopathological characteristics of this cohort stratified by BMI are summarized in Supplementary Table S3. The study selection process is illustrated in Figure 1.

The key characteristics of the included studies are provided in Table 1. The Newcastle-Ottawa Scale scores for each study are detailed in Supplementary Table S2. The studies were published between 2008 and 2026 and conducted across multiple regions, including North America, Europe, Asia, and the Middle East. A total of 40,501 patients were included, with study sizes ranging from 92 to 16,823 participants. The majority of studies were retrospective, with one prospective cohort, and the studies exhibited high methodological quality, with Newcastle–Ottawa Scale scores between 7 and 9.

**Table 1:** Basic characteristics and the quality evaluation of the studies included.

Author	Year	Study period	Country	Study design	Sample size	NOS	Mean age (years)	Male (%)	Surgical approach	ISGPS definition	BMI cutoff (kg/m^2^)
Roger Noun	2008	1999–2006	Lebanon	Retrospective	92	7	62.9	59.6	Open	Yes	30
Timothy K. Williams	2009	2000–2007	USA	Retrospective	240	8	64.8	52.9	Open	Yes	25, 30
Matthew Benns	2009	1991–2008	USA	Retrospective	309	7	65.3	49.8	Open	No	30
Susan Tsai	2010	1995–2005	USA	Retrospective	795	9	66.8	50.0	Open	Unclear	25, 30
Saboor Khan	2010	1981–2007	USA	Retrospective	586	8	66.5	56.5	Open	Unclear	25, 30, 35
Monica Dandona	2011	1995–2009	USA	Retrospective	355	8	65.5	53.1	Open	Unclear	18.5, 25, 30
Sébastien Gaujoux	2012	2000–2005	USA	Retrospective	328	8	69.5	45.1	Open	No	30
Marco Del Chiaro	2013	2004–2010	Sweden	Retrospective	367	9	64.5	56.0	Open	No	25
Ayman El Nakeeb	2014	2000–2012	Egypt	Retrospective	471	8	52.9	40.7	Open	Yes (ISGPF)	25
Abhishek Mathur	2015	2000–2012	USA	Retrospective	228	7	66.8	52.1	Open	No	30
Awad Shamali	2017	2007–2015	UK	Retrospective	524	8	66.3	57.9	Open	Yes	30
Mark D. Girgis	2017	2011–2015	USA	Retrospective	474	8	66.4	53.5	Mixed	Yes	30
Tianyu Tang	2020	2015–2019	China	Retrospective	227	7	66.2	61.4	Mixed	Yes (POPF only)	18.5, 25
Si-Yi Zou	2020	2005–2016	China	Retrospective	707	8	60.7	65.4	Open	Yes (ISGPF)	18.5, 23, 25
E.H. Chang	2020	2010–2015	USA	Retrospective	3834	9	66.2	57.9	Unclear	No	30
Patrick Téoule	2020	1997–2018	Germany	Retrospective	152	9	68.0	50.0	Unclear	Yes	30
Konstantinos A. Zorbas	2021	2014–2016	USA	Retrospective	10,316	9	64.9	58.2	Unclear	Unclear	18.5, 25, 30, 35, 40
Courtney M. Lattimore	2021	2014–2018	USA	Retrospective	16,823	9	66.0	56.3	Unclear	Yes (ISGPS)	18.5, 30, 40
Jana Enderes	2022	2015–2021	Germany	Retrospective	211	7	67.4	60.2	Open	Yes	30
Anthony Di Gioia	2022	2010–2019	Italy	Retrospective	1865	8	64.2	56.8	Open	Yes	30
Tzu-Hui Wei	2023	2014–2021	Taiwan, China	Retrospective	462	8	65.0	55.8	Mixed	Yes	25
Emanuel Shapera	2024	2012–2021	USA	Prospective	505	9	68.2	59.2	Mixed	Unclear	18.5, 25, 30
Boram Lee	2025	2004–2022	South Korea	Retrospective	404	8	66.0	55.5	Unclear	No	25
Oleksandr Kvasivka	2026	2018–2024	Ukraine	Retrospective	106	7	NA	NA	Mixed	Yes	25
PUMCH	2025	2017–2023	China	Retrospective	120	8	59.6	56.5	Mixed	Yes	25, 30

Study Design: All studies were retrospective unless specified as prospective. NOS scores: Quality assessment for observational studies was performed using the Newcastle–Ottawa Scale. Scores ≥7 indicate high quality. Surgical Approach: “Open” indicates exclusively open surgery; “Mixed” indicates a combination of open and minimally invasive approaches; “Unclear” indicates that the surgical approach was not specified. ISGPS Definition: Indicates whether the study used the ISGPS or equivalent consensus definition for postoperative pancreatic fistula (POPF). “Yes” indicates use of ISGPS or equivalent criteria; “No” indicates use of alternative definition; “Unclear” indicates insufficient information to determine; specific notations (e.g., ISGPF, POPF only) indicate the definition used. BMI Cutoff: Values indicate the BMI thresholds used for stratification in the original study.

BMI, body mass index; ISGPS, international study group for pancreatic surgery; ISGPF, international study group for pancreatic fistula; NA, not available; NOS, Newcastle–Ottawa Scale; PUMCH, peking union medical college hospital.

BMI classification varied across studies, with most adopting thresholds aligned with conventional definitions of overweight (≥25 kg/m^2^) and obesity (≥30 kg/m^2^), although additional cut-offs (e.g., 18.5, 23, 35, and 40 kg/m^2^) were used in some studies. For the present analysis, BMI categories were harmonized into three groups (<25, 25–30, and ≥30 kg/m^2^) to enable consistent comparisons. Accordingly, both dichotomous analyses (BMI ≥25 vs. <25 kg/m^2^ and BMI ≥30 vs. <30 kg/m^2^) and graded analyses were performed.

Most procedures were performed using an open surgical approach, although several studies included mixed or minimally invasive techniques. Definitions of POPF were generally based on ISGPS or ISGPF criteria, although some studies did not clearly specify the definition.

In addition to the published studies, an institutional retrospective cohort from Peking Union Medical College Hospital, comprising 120 consecutive patients between 2017 and 2023, was incorporated using consistent eligibility criteria and outcome definitions.

### 3.2 BMI ≥25 kg/m^2^ vs. <25 kg/m^2^

Fourteen studies provided data to compare patients with a BMI of ≥25 kg/m^2^ and those with a BMI of <25 kg/m^2^ (Table 2).

**Table 2:** Meta-analysis of outcomes comparing BMI ≥25 vs. <25 kg/m^2^.

Characteristics	Studies	BMI ≥25/BMI <25 (Events/Total or n)	Effect estimate (95% CI)	*p*	Heterogeneity
1. Clinical Baseline Characteristics					
Age (years)	13	8,328 vs. 6,747	MD = −0.84 [−1.75, 0.08]	0.07	*I*^2^ = 59%, *p* = 0.003
Gender (male)	14	4,946/8,695 vs. 3,730/7,275	OR = 1.17 [1.01, 1.34]	0.03	*I*^2^ = 54%, *p* = 0.009
ASA class III–IV	9	5,586/7,495 vs. 3,802/5,519	OR = 0.96 [0.77, 1.21]	0.75	*I*^2^ = 64%, *p* = 0.005
Soft pancreatic texture	6	2,655/4,744 vs. 1,777/3,672	OR = 1.43 [1.31, 1.57]	<0.00001	*I*^2^ = 21%, *p* = 0.28
Diabetes	6	1,957/6,914 vs. 1,117/4,965	OR = 0.98 [0.62, 1.56]	0.94	*I*^2^ = 84%, *p* < 0.0001
Hypertension	4	3,865/6,548 vs. 1,896/4,519	OR = 1.86 [1.10, 3.16]	0.02	*I*^2^ = 80%, *p* = 0.002
Weight loss	5	1,264/7,385 vs. 1,464/5,033	OR = 0.69 [0.52, 0.91]	0.009	*I*^2^ = 79%, *p* = 0.0007
Preoperative biliary drainage	5	254/561 vs. 656/1,486	OR = 0.85 [0.56, 1.29]	0.44	*I*^2^ = 70%, *p* = 0.009
Dilation of the pancreatic duct	5	3,519/5,424 vs. 2,858/4,385	OR = 0.82 [0.62, 1.07]	0.14	*I*^2^ = 61%, *p* = 0.04
Preoperative jaundice	4	3,275/6,940 vs. 2,441/4,837	OR = 0.97 [0.75, 1.27]	0.83	*I*^2^ = 53%, *p* = 0.10
Preoperative albumin (g/dL)	6	6,882 vs. 5,197	MD = 0.06 [0.04, 0.08]	<0.00001	*I*^2^ = 37%, *p* = 0.16
Preoperative bilirubin (*μ*mol/L)	3	186 vs. 632	MD = 2.34 [0.91, 3.77]	0.001	*I*^2^ = 0%, *p* = 0.81
CA19-9 (U/mL)	4	274 vs. 937	MD = 29.03 [−7.49, 65.56]	0.12	*I*^2^ = 0%, *p* = 0.47
Preoperative CEA (ug/mL)	4	274 vs. 938	MD = -0.65 [−2.40, 1.09]	0.46	*I*^2^ = 81%, *p* = 0.001
2. Tumor Pathological Characteristics					
Advanced tumor stage (III–IV)	4	480/768 vs. 664/944	OR = 0.92 [0.71, 1.21]	0.56	*I*^2^ = 28%, *p* = 0.24
Tumor size (cm)	8	1,598 vs. 2,032	MD = −0.00 [−0.04, 0.03]	0.77	*I*^2^ = 36%, *p* = 0.14
Perineural invasion	4	415/615 vs. 744/1,282	OR = 1.12 [0.87, 1.44]	0.38	*I*^2^ = 28%, *p* = 0.24
Vascular resection	3	1,037/6,700 vs. 840/4,688	OR = 0.86 [0.61, 1.21]	0.38	*I*^2^ = 55%, *p* = 0.11
R0 resection	7	907/1,301 vs. 1,014/1,563	OR = 1.17 [0.97, 1.40]	0.10	*I*^2^ = 40%, *p* = 0.13
Malignant pathology	5	5,571/6,853 vs. 4,229/5,109	OR = 0.94 [0.85, 1.04]	0.23	*I*^2^ = 13%, *p* = 0.33
Lymph node metastasis	7	721/1,274 vs. 919/1,503	OR = 0.89 [0.75, 1.06]	0.18	*I*^2^ = 0%, *p* = 0.93
3. Perioperative Operative Outcomes					
Surgical time (minutes)	12	8,122 vs. 6,598	MD = 15.77 [14.29, 17.26]	<0.00001	*I*^2^ = 43%, *p* = 0.06
Estimated blood loss (mL)	12	2,106 vs. 3,093	MD = 108.17 [48.74, 167.60]	0.0004	*I*^2^ = 94%, *p* < 0.00001
Number of dissected lymph nodes	4	678 vs. 1,389	MD = 0.16 [−1.12, 1.44]	0.81	*I*^2^ = 83%, *p* = 0.0006
Intraoperative blood transfusion	2	45/195 vs. 150/150	OR = 1.25 [0.46, 3.38]	0.66	*I*^2^ = 85%, *p* = 0.01
4. Postoperative Short-term Outcomes					
Pancreatic fistula (≥ Grade B)	9	1,540/7,459 vs. 689/6,110	OR = 2.49 [1.92, 3.23]	<0.00001	*I*^2^ = 48%, *p* = 0.05
Perioperative mortality	10	222/7,836 vs. 226/6,182	OR = 1.19 [0.75, 1.88]	0.45	*I*^2^ = 52%, *p* = 0.03
Wound infection	6	1,682/6,962 vs. 805/5,074	OR = 1.51 [1.38, 1.66]	<0.00001	*I*^2^ = 0%, *p* = 0.85
Delayed gastric emptying	7	1,273/7,035 vs. 748/5,633	OR = 1.39 [1.25, 1.54]	<0.00001	*I*^2^ = 30%, *p* = 0.20
Hemorrhage	5	48/860 vs. 73/2,103	OR = 1.61 [1.10, 2.34]	0.01	*I*^2^ = 0%, *p* = 0.57
Postoperative complications	5	4,096/6,851 vs. 2,317/4,613	OR = 1.33 [1.23, 1.43]	<0.00001	*I*^2^ = 47%, *p* = 0.11
Clavien–Dindo ≥ III	6	148/849 vs. 198/1,460	OR = 1.46 [1.14, 1.87]	0.003	*I*^2^ = 49%, *p* = 0.08
Bile leakage	4	26/458 vs. 54/1,205	OR = 1.43 [0.88, 2.31]	0.15	*I*^2^ = 0%, *p* = 0.85
Reoperation	3	21/317 vs. 37/979	OR = 1.86 [1.06, 3.24]	0.03	*I*^2^ = 18%, *p* = 0.30
Postoperative hospital stay (days)	11	8,253 vs. 6,717	MD = 0.96 [−0.45, 2.38]	0.18	*I*^2^ = 99%, *p* < 0.00001
5. Long-term Survival Outcomes					
Overall survival (OS)	6	942 vs. 1,226	HR = 0.98 [0.83, 1.14]	0.76	*I*^2^ = 52%, *p* = 0.06

Data presentation: For binary outcomes, data are presented as *events/total in BMI ≥25 group vs. events/total in BMI <25 group*. For continuous outcomes, data are presented as sample size (n) in BMI ≥25 group vs. sample size in BMI <25 group. For overall survival, hazard ratios (HR) are presented; for other outcomes, odds ratios (OR) or mean differences (MD) are presented as indicated. Heterogeneity: *I*^2^ values and corresponding *p*-values from Cochran's *Q* test are reported. Random-effects models were applied when *I*^2^ > 50%; otherwise, fixed-effects models were used.

BMI, body mass index; CI, confidence interval; HR, hazard ratio; MD, mean difference; OR, odds ratio.

#### 3.2.1 Baseline and tumor characteristics

No significant age difference was observed between the groups (MD = −0.84 years, 95% CI: −1.75 to 0.08, *P* = 0.07). Patients with a BMI of ≥25 kg/m^2^ were more frequently male (OR = 1.17, 95% CI: 1.01–1.34, *P* = 0.03) and had a greater prevalence of soft pancreatic texture (OR = 1.43, 95% CI: 1.31–1.57, *P* < 0.00001) and hypertension (OR = 1.86, 95% CI: 1.10–3.16, *P* = 0.02).

Preoperative weight loss was less frequent (OR = 0.69, 95% CI: 0.52–0.91, *P* = 0.009), while albumin (MD = 0.06 g/dL, 95% CI: 0.04–0.08, *P* < 0.00001) and bilirubin levels (MD = 2.34 μmol/L, 95% CI: 0.91–3.77, *P* = 0.001) were slightly higher. Other baseline variables, including ASA score, diabetes, and pancreatic duct dilation, showed no significant differences. Tumor-related characteristics were comparable between groups.

#### 3.2.2 Operative outcomes

**Figure 2:**
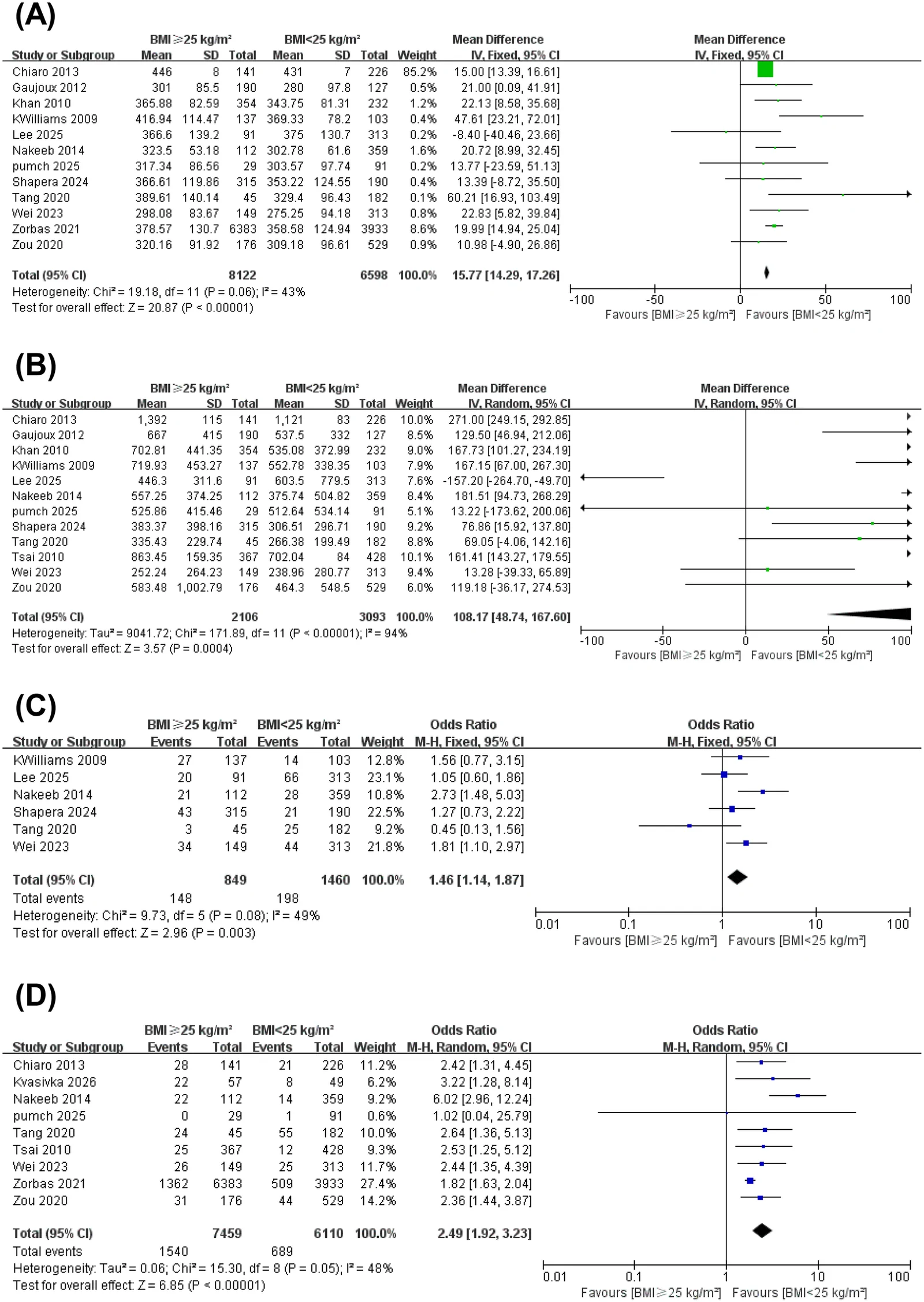
Forest plots comparing perioperative outcomes between BMI ≥25 and <25 kg/m^2^. Forest plots showing the association between BMI ≥25 kg/m^2^ and perioperative outcomes after pancreaticoduodenectomy. **(A)** Operative time; **(B)** Estimated blood loss; **(C)** Major complications (Clavien–Dindo grade ≥ III); **(D)** Postoperative pancreatic fistula (grade B/C). Effect estimates are presented as odds ratios (ORs) for dichotomous outcomes and mean differences (MDs) for continuous outcomes, with 95% confidence intervals (CIs). A random-effects model was applied when statistical heterogeneity was significant (*I*^2^ > 50%); otherwise, a fixed-effects model was used.

Patients with BMI ≥25 kg/m^2^ had significantly longer operative time (MD = 15.77 min, 95% CI: 14.29–17.26, *P* < 0.00001; *I*^2^ = 43%, Figure 2A) and increased estimated blood loss (MD = 108.17 mL, 95% CI: 48.74–167.60, *P* = 0.0004; *I*^2^ = 94%, Figure 2B).

#### 3.2.3 Postoperative outcomes

BMI ≥25 kg/m^2^ was linked to a notably higher risk of postoperative morbidity. The rates of overall complications (OR = 1.33, 95% CI: 1.23–1.43, *P* < 0.00001) and major complications (Clavien–Dindo ≥ III; OR = 1.46, 95% CI: 1.14–1.87, *P* = 0.003, Figure 2C) were elevated.

Specific complications were also more frequent, including clinically relevant POPF (≥ Grade B) (OR = 2.49, 95% CI: 1.92–3.23, *P* < 0.0001; *I*^2^ = 48%, Figure 2D), delayed gastric emptying (OR = 1.39, 95% CI: 1.25–1.54, *P* < 0.00001), wound infection (OR = 1.51, 95% CI: 1.38–1.66, *P* < 0.00001), postoperative hemorrhage (OR = 1.61, 95% CI: 1.10–2.34, *P* = 0.01), and reoperation (OR = 1.86, 95% CI: 1.06–3.24, *P* = 0.03).

There were no notable differences observed in perioperative mortality (OR = 1.19, 95% CI: 0.75–1.88, *P* = 0.45; *I*^2^ = 52%) or postoperative hospital stay, with substantial heterogeneity observed across studies (MD = 0.96 days, 95% CI: −0.45 to 2.38, *P* = 0.18; *I*^2^ = 99%).

#### 3.2.4 Long-term outcomes

No meaningful association was found between BMI and overall survival (HR = 0.98, 95% CI: 0.83–1.14, *P* = 0.76).

Further perioperative outcomes for this comparison are shown in Supplementary Figure S2.

### 3.3 BMI ≥30 kg/m^2^ vs. <30 kg/m^2^

Seventeen studies were included in this comparison (Table 3).

**Table 3:** Meta-analysis of outcomes comparing BMI ≥30 vs. <30 kg/m^2^.

Characteristics	Studies	BMI ≥30/BMI <30 (Events/Total or n)	Effect estimate (95% CI)	*p*	Heterogeneity
1. Clinical Baseline Characteristics					
Age (years)	16	9,282 vs. 27,199	MD = −2.43 [−2.68, −2.19]	<0.00001	*I*^2^ = 45%, *p* = 0.03
Gender (male)	17	4,757/9,316 vs. 15,254/27,760	OR = 0.86 [0.82, 0.90]	<0.00001	*I*^2^ = 0%, *p* = 0.68
ASA class III–IV	8	6,318/7,902 vs. 15,860/22,878	OR = 1.64 [1.42, 1.90]	<0.00001	*I*^2^ = 54%, *p* = 0.04
Soft pancreatic texture	5	3,201/5,737 vs. 6,916/15,182	OR = 1.42 [1.34, 1.51]	<0.00001	*I*^2^ = 0%, *p* = 0.91
Diabetes	9	2,773/8,525 vs. 5,537/24,949	OR = 1.65 [1.57, 1.75]	<0.00001	*I*^2^ = 0%, *p* = 0.84
Hypertension	6	5,516/8,443 vs. 11,363/23,162	OR = 1.95 [1.85, 2.05]	<0.00001	*I*^2^ = 11%, *p* = 0.34
Weight loss	9	1,088/8,785 vs. 5,734/26,136	OR = 0.62 [0.54, 0.70]	<0.00001	*I*^2^ = 51%, *p* = 0.04
Preoperative biliary drainage	5	138/1,608 vs. 1,222/2,568	OR = 0.63 [0.50, 0.79]	<0.0001	*I*^2^ = 22%, *p* = 0.27
Dilation of the pancreatic duct	3	3,637/14,871 vs. 11,234/15,826	OR = 0.72 [0.68, 0.77]	<0.00001	*I*^2^ = 0%, *p* = 0.76
Preoperative jaundice	4	1,401/6,437 vs. 5,036/10,168	OR = 0.87 [0.80, 0.94]	0.0007	*I*^2^ = 21%, *p* = 0.28
History of smoking	6	1,286/6,297 vs. 5,011/24,800	OR = 0.78 [0.69, 0.88]	<0.0001	*I*^2^ = 50%, *p* = 0.08
COPD	6	411/1,404 vs. 993/23,162	OR = 1.14 [1.01, 1.29]	0.03	*I*^2^ = 6%, *p* = 0.38
Congestive heart failure	3	40/103 vs. 63/22,421	OR = 1.74 [1.17, 2.59]	0.006	*I*^2^ = 0%, *p* = 0.48
Preoperative steroid use	3	188/742 vs. 554/22,421	OR = 0.92 [0.78, 1.09]	0.36	*I*^2^ = 0%, *p* = 0.93
2. Tumor Pathological Characteristics					
Tumor size (cm)	7	557 vs. 2,391	MD = −0.04 [−0.21, 0.14]	0.66	*I*^2^ = 85%, *p* < 0.00001
Vascular resection	5	488/3,096 vs. 1,636/9,813	OR = 0.85 [0.76, 0.96]	0.006	*I*^2^ = 0%, *p* = 0.80
R0 resection	6	439/599 vs. 1,880/2,800	OR = 1.04 [0.77, 1.42]	0.78	*I*^2^ = 53%, *p* = 0.06
Malignant pathology	4	2,338/2,950 vs. 6,858/8,341	OR = 0.83 [0.74, 0.92]	0.0004	*I*^2^ = 27%, *p* = 0.25
Lymph node metastasis	6	251/1,470 vs. 1,219/2,105	OR = 0.92 [0.74, 1.15]	0.48	*I*^2^ = 0%, *p* = 0.51
3. Perioperative Operative Outcomes					
Surgical time (minutes)	14	8,347 vs. 24,295	MD = 17.62 [11.21, 24.04]	<0.00001	*I*^2^ = 62%, *p* = 0.001
Estimated blood loss (mL)	12	1,092 vs. 5,114	MD = 113.21 [41.02, 185.41]	0.002	*I*^2^ = 89%, *p* < 0.00001
Number of dissected lymph nodes	3	291 vs. 2,580	MD = 1.17 [−0.94, 3.28]	0.28	*I*^2^ = 67%, *p* = 0.05
Intraoperative blood transfusion	5	57/296 vs. 239/2,467	OR = 1.16 [0.82, 1.63]	0.41	*I*^2^ = 0%, *p* = 0.66
4. Postoperative Short-term Outcomes					
Pancreatic fistula (≥ Grade B)	10	1,855/8,006 vs. 3,545/23,477	OR = 1.74 [1.63, 1.85]	<0.00001	*I*^2^ = 0%, *p* = 0.85
Perioperative mortality	11	219/651 vs. 432/25,888	OR = 1.55 [1.31, 1.83]	<0.00001	*I*^2^ = 0%, *p* = 0.63
Wound infection	11	2,504/7,653 vs. 5,149/25,711	OR = 1.50 [1.42, 1.59]	<0.00001	*I*^2^ = 0%, *p* = 0.90
Delayed gastric emptying	7	1,388/4,660 vs. 3,272/20,882	OR = 1.19 [1.11, 1.27]	<0.00001	*I*^2^ = 0%, *p* = 0.79
Hemorrhage	5	234/957 vs. 723/3,377	OR = 1.03 [0.87, 1.22]	0.73	*I*^2^ = 0%, *p* = 0.74
Postoperative complications	9	3,814/13,711 vs. 9,897/22,743	OR = 1.31 [1.25, 1.39]	<0.00001	*I*^2^ = 0%, *p* = 0.58
Clavien–Dindo ≥ III	4	105/350 vs. 245/1,070	OR = 1.50 [1.13, 1.98]	0.005	*I*^2^ = 0%, *p* = 0.76
Pneumonia	4	250/1,110 vs. 860/16,645	OR = 1.13 [0.97, 1.31]	0.11	*I*^2^ = 0%, *p* = 0.71
Reoperation	5	295/1,107 vs. 812/14,625	OR = 1.17 [1.01, 1.34]	0.03	*I*^2^ = 0%, *p* = 0.99
Re-admission	3	1,414/4,594 vs. 3,180/20,224	OR = 1.25 [1.17, 1.34]	<0.00001	*I*^2^ = 0%, *p* = 0.86
Intra-abdominal abscess	3	55/461 vs. 406/1,964	OR = 1.55 [1.11, 2.16]	0.01	*I*^2^ = 4%, *p* = 0.35
Wound dehiscence	3	144/395 vs. 251/22,421	OR = 1.58 [1.29, 1.94]	<0.0001	*I*^2^ = 0%, *p* = 0.89
Postoperative hospital stay (days)	11	3,679 vs. 12,238	MD = 1.06 [0.40, 1.71]	0.002	*I*^2^ = 84%, *p* < 0.00001
5. Long-term Survival Outcomes					
Overall survival (OS)	6	457 vs. 1,868	HR = 1.04 [0.94, 1.16]	0.46	*I*^2^ = 46%, *p* = 0.10

Data presentation: For binary outcomes, data are presented as *events/total in BMI ≥30 group vs. events/total in BMI <30 group*. For continuous outcomes, data are presented as sample size (n) in BMI ≥30 group vs. sample size in BMI <30 group. For overall survival, hazard ratios (HR) are presented; for other outcomes, odds ratios (OR) or mean differences (MD) are presented as indicated. Heterogeneity: *I*^2^ values and corresponding *p*-values from Cochran's *Q* test are reported. Random-effects models were applied when *I*^2^ > 50%; otherwise, fixed-effects models were used.

BMI, body mass index; CI, confidence interval; HR, hazard ratio; MD, mean difference; OR, odds ratio.

#### 3.3.1 Baseline characteristics

Patients with BMI ≥30 kg/m^2^ were notably younger (MD = −2.43 years, 95% CI: −2.68 to −2.19, *P* < 0.00001) and had a greater burden of comorbidities, including higher rates of diabetes (OR = 1.65, 95% CI: 1.57–1.75, *P* < 0.00001), hypertension (OR = 1.95, 95% CI: 1.85–2.05, *P* < 0.00001), and an elevated ASA classification (OR = 1.64, 95% CI: 1.42–1.90, *P* < 0.00001).

A higher prevalence of soft pancreatic texture (OR = 1.42, 95% CI: 1.34–1.51, *P* < 0.00001) and a lower frequency of preoperative weight loss (OR = 0.62, 95% CI: 0.54–0.70, *P* < 0.00001) were also observed.

Overall, obese patients presented with a higher comorbidity burden and less favorable physiological profiles.

#### 3.3.2 Tumor-related characteristics

Tumor characteristics were largely comparable between groups. Obese patients were less likely to undergo vascular resection (OR = 0.85, 95% CI: 0.76–0.96, *P* = 0.006), while no significant differences were observed in tumor size, R0 resection rate, or lymph node metastasis.

#### 3.3.3 Operative outcomes

Obesity was associated with increased operative complexity, reflected by longer operative time (MD = 17.62 min, 95% CI: 11.21–24.04, *P* < 0.00001; *I*^2^ = 62%) and greater estimated blood loss (MD = 113.21 mL, 95% CI: 41.02–185.41, *P* = 0.002; *I*^2^ = 89%). No significant difference was observed in intraoperative blood transfusion.

#### 3.3.4 Postoperative outcomes

**Figure 3:**
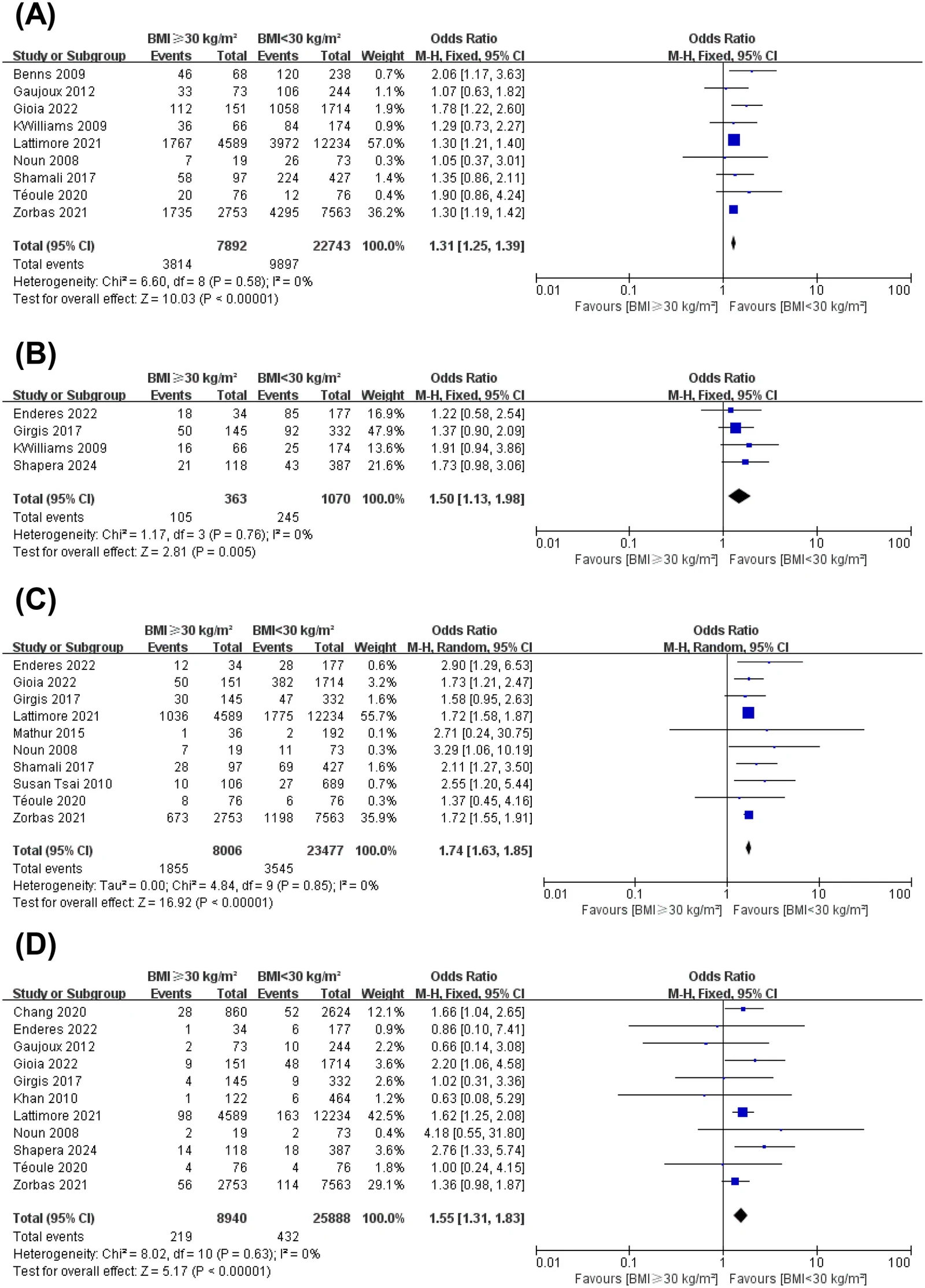
Forest plots comparing perioperative outcomes between BMI ≥30 and <30 kg/m^2^. Forest plots showing the association between BMI ≥30 kg/m^2^ and perioperative outcomes after pancreaticoduodenectomy. **(A)** Overall postoperative complications; **(B)** major complications (Clavien–Dindo grade ≥ III); **(C)** postoperative pancreatic fistula (grade B/C); **(D)** perioperative mortality. Effect estimates are presented as odds ratios (ORs) with 95% confidence intervals (CIs). A random-effects model was applied when heterogeneity was significant (*I*^2^ > 50%); otherwise, a fixed-effects model was used.

BMI ≥30 kg/m^2^ was associated with significantly increased risks of overall complications (OR = 1.31, 95% CI: 1.25–1.39, *P* < 0.00001, Figure 3A) and major complications (Clavien–Dindo ≥ III; OR = 1.50, 95% CI: 1.13–1.98, *P* = 0.005, Figure 3B).

The incidence of clinically relevant POPF (≥ Grade B) was also higher (OR = 1.74, 95% CI: 1.63–1.85, *P* < 0.0001; *I*^2^ = 0%, Figure 3C), along with increased risks of wound infection (OR = 1.50, 95% CI: 1.42–1.59, *P* < 0.00001), delayed gastric emptying (OR = 1.19, 95% CI: 1.11–1.27, *P* < 0.00001), and reoperation (OR = 1.17, 95% CI: 1.01–1.34, *P* = 0.03).

Importantly, perioperative mortality was significantly higher in patients with BMI ≥30 kg/m^2^ (OR = 1.55, 95% CI: 1.31–1.83, *P* < 0.00001, Figure 3D), whereas no significant difference was observed in postoperative hemorrhage.

Postoperative hospital stay was also prolonged (MD = 1.06 days, 95% CI: 0.40–1.71, *P* = 0.002).

These findings indicate an overall increase in postoperative morbidity associated with obesity.

#### 3.3.5 Long-term outcomes

Obesity was not significantly associated with overall survival (HR = 1.04, 95% CI: 0.94–1.16, *P* = 0.46).

Further perioperative outcomes for this comparison are shown in Supplementary Figure S3.

### 3.4 Graded pairwise comparisons among BMI categories

As shown in Supplementary Table S4, exploratory comparisons across three BMI categories (<25, 25–30, and ≥30 kg/m^2^), each supported by at least three studies, suggested possible graded and threshold patterns across selected outcomes.

**Figure 4:**
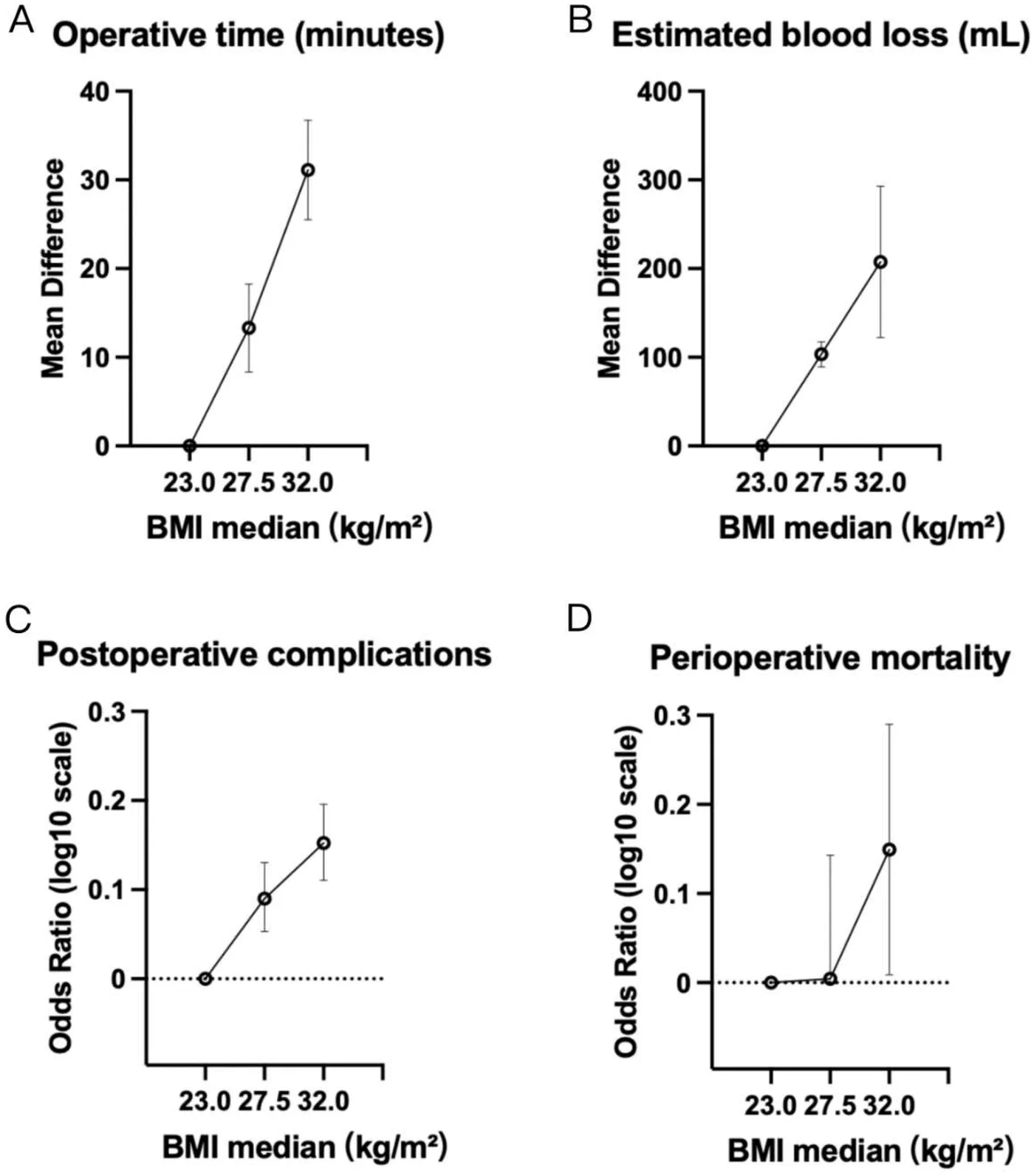
Trends in perioperative outcomes across BMI categories (<25, 25–30, ≥30 kg/m^2^). Line plots illustrating trends in perioperative outcomes across BMI categories (<25, 25–30, ≥30 kg/m^2^), using the lowest BMI category (<25 kg/m^2^) as the reference. **(A)** Operative time (mean difference, minutes); **(B)** estimated blood loss (mean difference, mL); **(C)** postoperative complications; **(D)** perioperative mortality. For dichotomous outcomes **(C,D)**, effect estimates are expressed as odds ratios (ORs) on a log₁₀ scale. Error bars represent 95% confidence intervals (CIs). These plots are based on pooled estimates from pairwise meta-analyses and are intended to illustrate incremental patterns across BMI categories rather than formal dose–response relationships.

Operative parameters indicated a clear graded relationship. Operative time (Figure 4A) and estimated blood loss (Figure 4B) both showed a gradual increase with higher BMI, indicating greater surgical complexity. Compared with patients with BMI <25 kg/m^2^, those with BMI 25–30 kg/m^2^ had a mean increase of 13.31 min in operative time, which further increased to 31.12 min in patients with BMI ≥30 kg/m^2^ (*P* < 0.00001). A similar pattern was observed for estimated blood loss, with mean differences of 103.40 mL and 207.48 mL in the overweight and obese groups, respectively.

Overall postoperative complications showed a graded pattern, with odds ratios rising from 1.23 (95% CI: 1.13–1.35) in the overweight group to 1.42 (95% CI: 1.29–1.57) in the obese group (Figure 4C). In contrast, severe outcomes exhibited a threshold pattern. Perioperative mortality was not significantly different between patients with BMI <25 kg/m^2^ and those with BMI 25–30 kg/m^2^ (OR = 1.01, *P* = 0.95). Nevertheless, it was notably higher in patients with BMI ≥30 kg/m^2^ compared to those with BMI <25 kg/m^2^ (OR = 1.41, *P* = 0.04) (Figure 4D).

Tumor-related characteristics, including tumor size, R0 resection rate, and lymph node metastasis, were comparable across BMI categories (Supplementary Table S4), suggesting that differences in perioperative outcomes were unlikely to be attributable to tumor burden.

### 3.5 Heterogeneity, sensitivity analysis and publication bias

Substantial heterogeneity was observed for several outcomes, particularly estimated blood loss, POPF, and postoperative hospital stay. Subgroup analyses stratified by POPF definition, surgical approach, geographic region, and publication year are presented in Supplementary Table S5. Significant subgroup differences were confined to the BMI ≥25 kg/m^2^ comparison, whereas no significant subgroup differences were observed in the BMI ≥30 kg/m^2^ comparison. Surgical-approach subgroup analyses showed no significant differences between open-only cohorts and cohorts including minimally invasive pancreaticoduodenectomy (MIPD) for the primary outcomes (Supplementary Table S6). Exploratory meta-regression analyses suggested that several study-level factors, including publication year, surgical technique, and geographic region, were individually associated with heterogeneity in blood loss. For POPF, surgical technique, sample size, and age were identified as potential sources of heterogeneity, while age was associated with variation in mortality and hospital stay (all *P* < 0.05).

Leave-one-out sensitivity analyses showed that sequential exclusion of individual studies did not alter the direction or overall interpretation of the pooled estimates for the primary outcomes. The associations with clinically relevant POPF remained statistically significant in both BMI comparisons. For major complications, the pooled estimate remained stable in the BMI ≥30 kg/m^2^ comparison, whereas its statistical significance varied in some iterations in the BMI ≥25 kg/m^2^ comparison, although the direction of effect was unchanged. Exclusion of the PUMCH cohort did not materially alter the pooled estimates, indicating that the main findings were not driven by the institutional cohort (Supplementary Figure S4, Supplementary Table S7).

Publication bias was assessed using funnel plots and Egger's regression test for outcomes including ≥10 studies. No significant publication bias was detected for most eligible outcomes in both BMI ≥25 kg/m^2^ and BMI ≥30 kg/m^2^ comparisons (all Egger's test *P* > 0.05). Funnel plots for primary outcomes are shown in Supplementary Figure S5. For outcomes with fewer than 10 studies, publication bias was not formally assessed due to limited statistical power.

## 4 Discussion

This systematic review and meta-analysis, comprising 24 published studies and one institutional cohort, examined the influence of BMI on perioperative outcomes in patients undergoing PD. The key findings were as follows: BMI ≥25 kg/m^2^ correlated with increased operative time, higher estimated blood loss, and a greater likelihood of postoperative morbidity, especially clinically significant POPF. Major complications and reoperation were also increased in this group. In contrast, BMI ≥30 kg/m^2^ was linked to an increased risk of perioperative mortality. No significant association was observed between BMI and long-term overall survival, although the available evidence was limited.

The increased operative complexity observed in patients with BMI ≥25 kg/m^2^ is likely related to greater visceral adiposity and a higher prevalence of soft pancreatic texture, which may complicate surgical exposure and reconstruction (16). Importantly, BMI does not distinguish between visceral adipose tissue, subcutaneous fat, and skeletal muscle mass. Body-composition measures, including visceral fat area and the visceral adipose tissue-to-skeletal muscle ratio, may therefore provide a more direct assessment of surgical risk than BMI alone (13, 16). A higher visceral fat burden may impair operative exposure, increase technical difficulty, and prolong operative time, whereas reduced skeletal muscle mass may reflect poorer physiological reserve and greater vulnerability to postoperative complications. Visceral adiposity has been independently associated with prolonged operative time, increased intraoperative blood loss, and greater technical difficulty in achieving secure pancreaticojejunal anastomosis (13). These factors align with the observed increases in operative time, blood loss, and postoperative complications, including moderate events (e.g., delayed gastric emptying, wound infection) and severe events such as clinically relevant POPF, major complications, and reoperation (5–7). Specifically, meta-analyses have demonstrated that patients with elevated BMI (≥25 kg/m^2^ or ≥30 kg/m^2^) have a nearly two-fold increased risk of clinically relevant POPF (OR = 1.96–1.97) and a significantly higher risk of overall major complications (OR = 1.85) (36, 37). Notably, the absence of a significant increase in perioperative mortality suggests that moderate elevations in BMI primarily affect technical difficulty and postoperative morbidity, without extending to mortality (11). Subgroup analyses suggested that differences in POPF definition and study setting may partly contribute to variation in the BMI ≥25 kg/m^2^ comparison. However, neither surgical approach nor publication era consistently modified the association, and no significant subgroup differences were identified in the BMI ≥30 kg/m^2^ comparison. These findings should nevertheless be considered exploratory because several strata included a limited number of studies.

Patients with BMI ≥30 kg/m^2^ exhibited a less favorable perioperative profile, characterized by a higher burden of comorbidities and reduced physiological reserve (10). In this group, elevated BMI was associated not only with increased operative complexity and postoperative complications but also with a higher risk of perioperative mortality. These findings support a threshold effect for mortality at BMI ≥30 kg/m^2^, beyond which the clinical impact extends from technical challenges to more severe adverse outcomes. Potential mechanisms include chronic low-grade inflammation (17, 18), impaired microvascular perfusion, and increased pancreatic fat infiltration (21), all of which have been linked to impaired healing and a higher risk of POPF.

A recent meta-analysis by Tai et al. reported that the association between BMI and postoperative mortality was attenuated in high-volume centers (33). This finding does not contradict our results; rather, it suggests that optimized perioperative management and institutional expertise may mitigate the adverse prognostic impact of obesity. Our findings should therefore be interpreted as the baseline risk associated with obesity in the overall PD population, which may be modifiable through enhanced perioperative care. Unfortunately, due to insufficient reporting of institutional surgical volume in the included studies, we were unable to perform a PD-specific subgroup analysis stratified by center volume. We acknowledge this as a limitation and suggest it as a direction for future research using individual patient-level data or multi-center registries that report center-level volume.

Although the *I*^2^ statistic for perioperative mortality was 0% in the BMI ≥30 comparison, indicating low statistical heterogeneity, this does not imply clinical homogeneity. Baseline perioperative mortality rates varied across the included studies, reflecting differences in healthcare systems, study periods, institutional expertise, and perioperative management. The low *I*^2^ value indicates that the relative effect of BMI on mortality was consistent across studies despite these differences in baseline risk.

Graded analyses across BMI categories further revealed that BMI-related risk patterns vary by outcome. Operative parameters, such as operative time and estimated blood loss, increased progressively with higher BMI, indicating a stepwise relationship between adiposity and surgical complexity (38). Perioperative mortality, however, followed a threshold pattern, with a marked increase observed only at BMI ≥30 kg/m^2^ (37, 39). Major complications were increased across BMI thresholds with comparable effect sizes, suggesting a graded rather than a strictly threshold-dependent association (11, 40). Collectively, these findings underscore that the impact of BMI is outcome-specific and highlight the value of stratified risk assessment over simple dichotomization. However, because several category-specific estimates were based on a limited number of studies, these findings should be considered exploratory and hypothesis-generating.

These results may help resolve inconsistencies in the existing literature. Studies that combine overweight and obese patients into a single high-BMI category may obscure the distinct impact of obesity, potentially contributing to neutral or conflicting findings (11, 37). By distinguishing between overweight (BMI 25–30 kg/m^2^) and obese (BMI ≥30 kg/m^2^) patients, the present analysis provides a more refined characterization of BMI-related risk patterns in PD.

From a clinical standpoint, BMI ≥30 kg/m^2^ may serve as a meaningful risk threshold for perioperative mortality (37). Patients in this subgroup may benefit from enhanced preoperative optimization, meticulous intraoperative technique to reduce POPF risk, and closer postoperative monitoring (3). For patients with BMI ≥25 kg/m^2^, the increased operative complexity and morbidity warrant careful perioperative planning, though the intensity of intervention may differ from that required in the obese subgroup. The apparent “obesity paradox” reported in some individual studies may reflect reverse causation, as lower BMI can indicate cancer-related weight loss or advanced disease. Lower BMI may also be associated with cachexia, sarcopenia, impaired nutritional status, and reduced physiological reserve, all of which may adversely affect long-term survival. In addition, differences in underlying disease indication, tumor biology, use of adjuvant therapy, and follow-up duration may confound the observed association between BMI and survival. The current evidence is therefore insufficient to support a protective effect of higher BMI on long-term survival after PD.

Several limitations must be recognized. First, all included studies were observational, predominantly retrospective, and therefore subject to selection bias and residual confounding. Second, BMI is an imperfect surrogate for adiposity and does not capture fat distribution, particularly visceral fat, which may be more directly relevant to surgical risk. Differences in the measurement and reporting of visceral fat and other body-composition indices precluded quantitative synthesis of these measures. Third, substantial heterogeneity was observed for several outcomes, which may reflect differences in patient populations, surgical techniques, and outcome definitions across studies. Although meta-regression suggested that factors such as surgical approach and geographic region may contribute to this heterogeneity, the findings should be interpreted with caution, considering the limitations of study-level analyses. Fourth, the lack of institutional volume data in the included studies precluded a volume-stratified subgroup analysis, which may have provided additional insights into whether the BMI-mortality association is modified by center expertise (33). Fifth, the BMI thresholds of 25 and 30 kg/m^2^ used in this study are based on WHO Western criteria. Given that nearly half of the included studies originated from Asia and the Middle East, where WHO recommends lower cut-offs (23 kg/m^2^ for overweight and 27.5 kg/m^2^ for obesity), the use of Western thresholds may have introduced some degree of misclassification in these populations. Finally, each overall survival analysis included only six studies and was affected by clinical and methodological heterogeneity; therefore, the survival findings should be interpreted with caution.

In conclusion, overweight (BMI ≥25 kg/m^2^) is primarily associated with increased operative complexity and moderate morbidity, whereas obesity (BMI ≥30 kg/m^2^) represents a distinct threshold associated with perioperative mortality. These findings support BMI-based risk stratification and suggest that individualized perioperative management should be particularly considered for patients with BMI ≥30 kg/m^2^.

## Data Availability

The raw data supporting the conclusions of this article will be made available by the authors, without undue reservation.
