# Supplementary material for: Graded and threshold effects of body mass index on perioperative outcomes after pancreaticoduodenectomy: a meta-analysis incorporating an institutional cohort
Source: Front Surg. 2026 Aug 21;13:1846295. doi: 10.3389/fsurg.2026.1846295 (PMC13575552; doi:10.3389/fsurg.2026.1846295)
Supplement: Supplementary file 1 [file Datasheet1.pdf]

frontiers

Supplementary Material

1 Supplementary Figures and Tables

1.1 Supplementary Figures

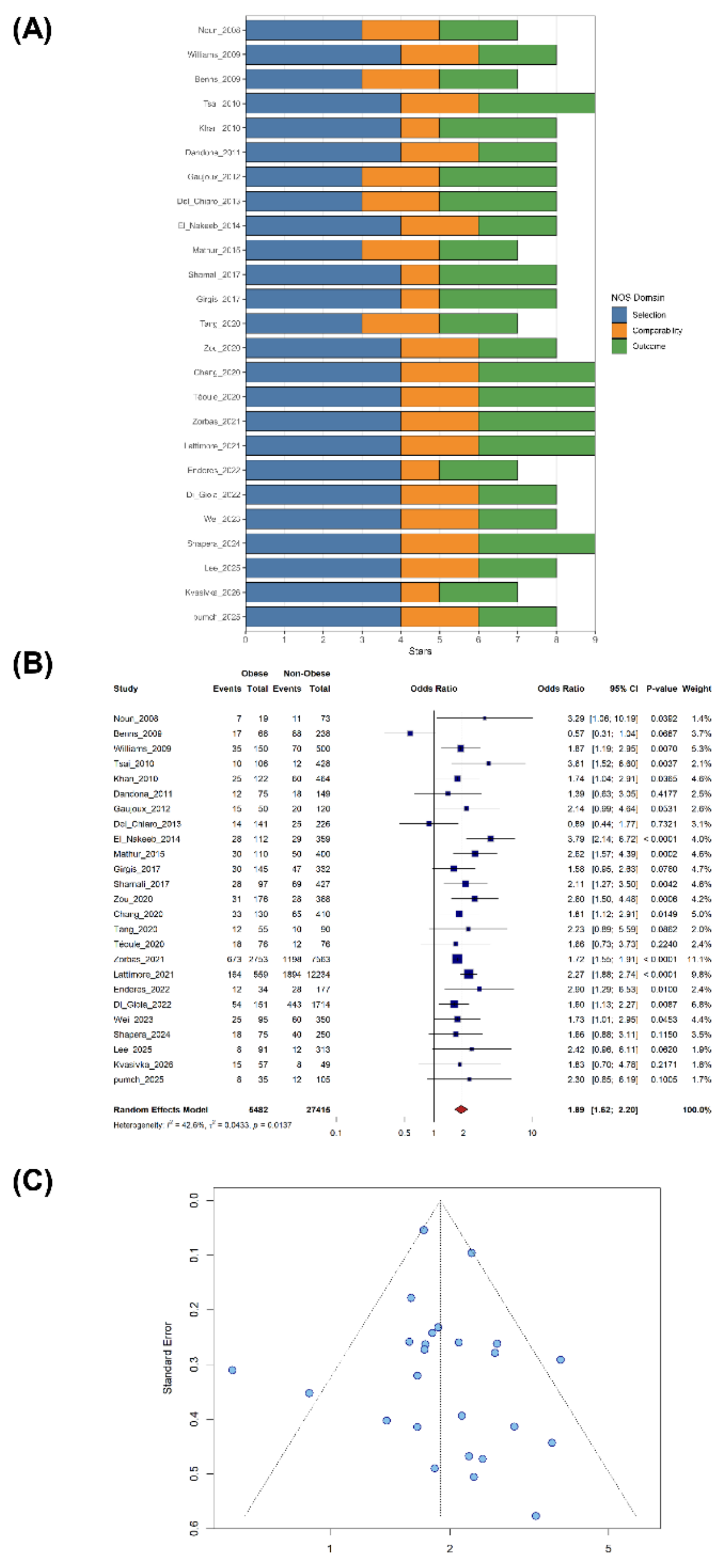

Figure S1. Methodological quality assessment of the included studies based on the Newcastle-Ottawa Scale (NOS), including (A) the quality summary, (B) quality-based subgroup analysis, (C) and associated publication bias evaluation.

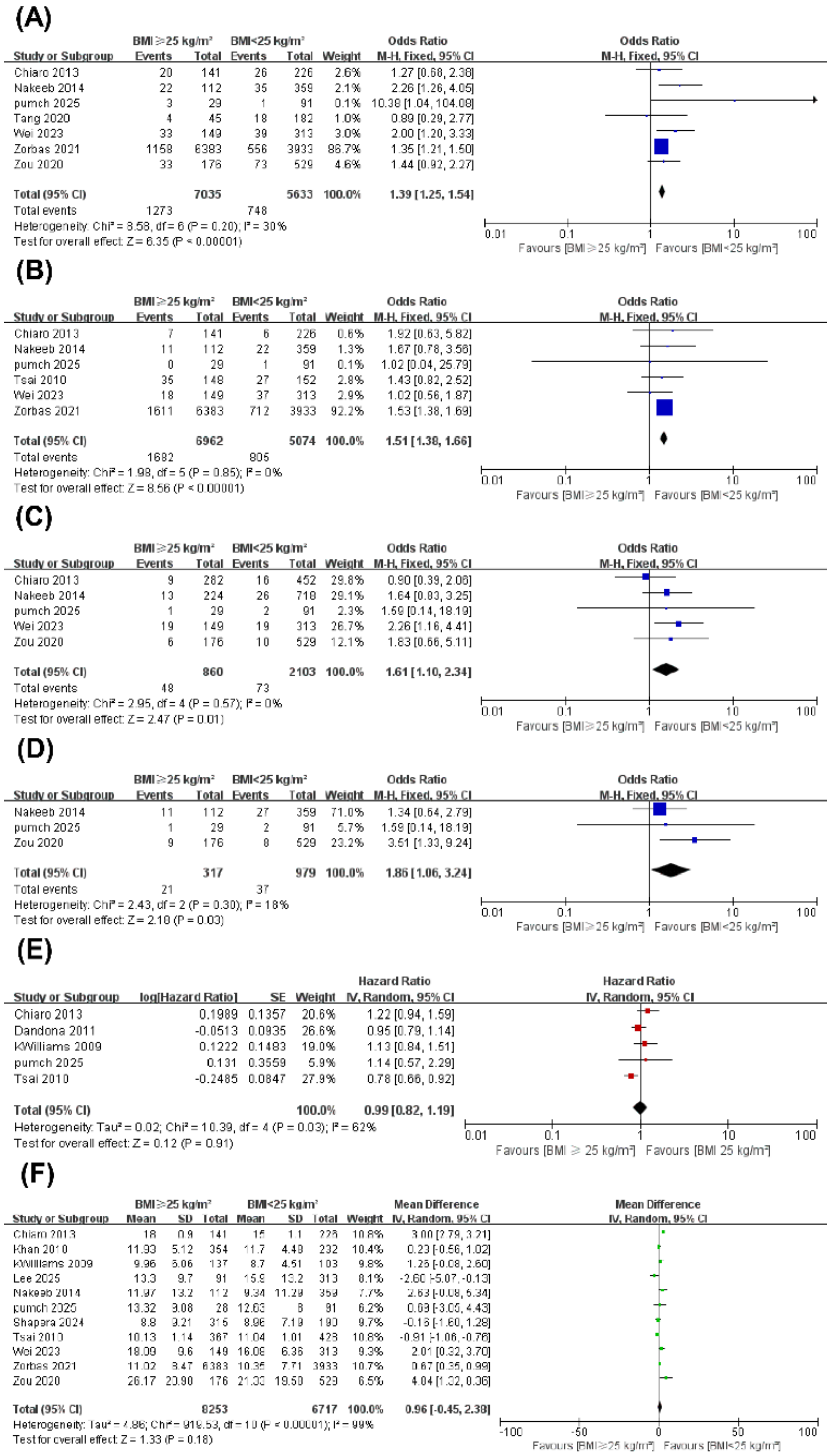

Figure S2. Forest plots showing the association between BMI ≥25 kg/m² and perioperative outcomes not presented in the main figures: (A) delayed gastric emptying; (B) wound infection; (C) postoperative hemorrhage; (D) reoperation rate; (E) overall survival (hazard ratio); (F) postoperative hospital stay (mean difference, days). Effect estimates are presented as odds ratios (ORs) for dichotomous outcomes and mean differences (MDs) for continuous outcomes, with 95% confidence intervals (CIs). Statistical models were applied according to the level of heterogeneity (random-effects model for I² >50%; fixed-effects model otherwise).

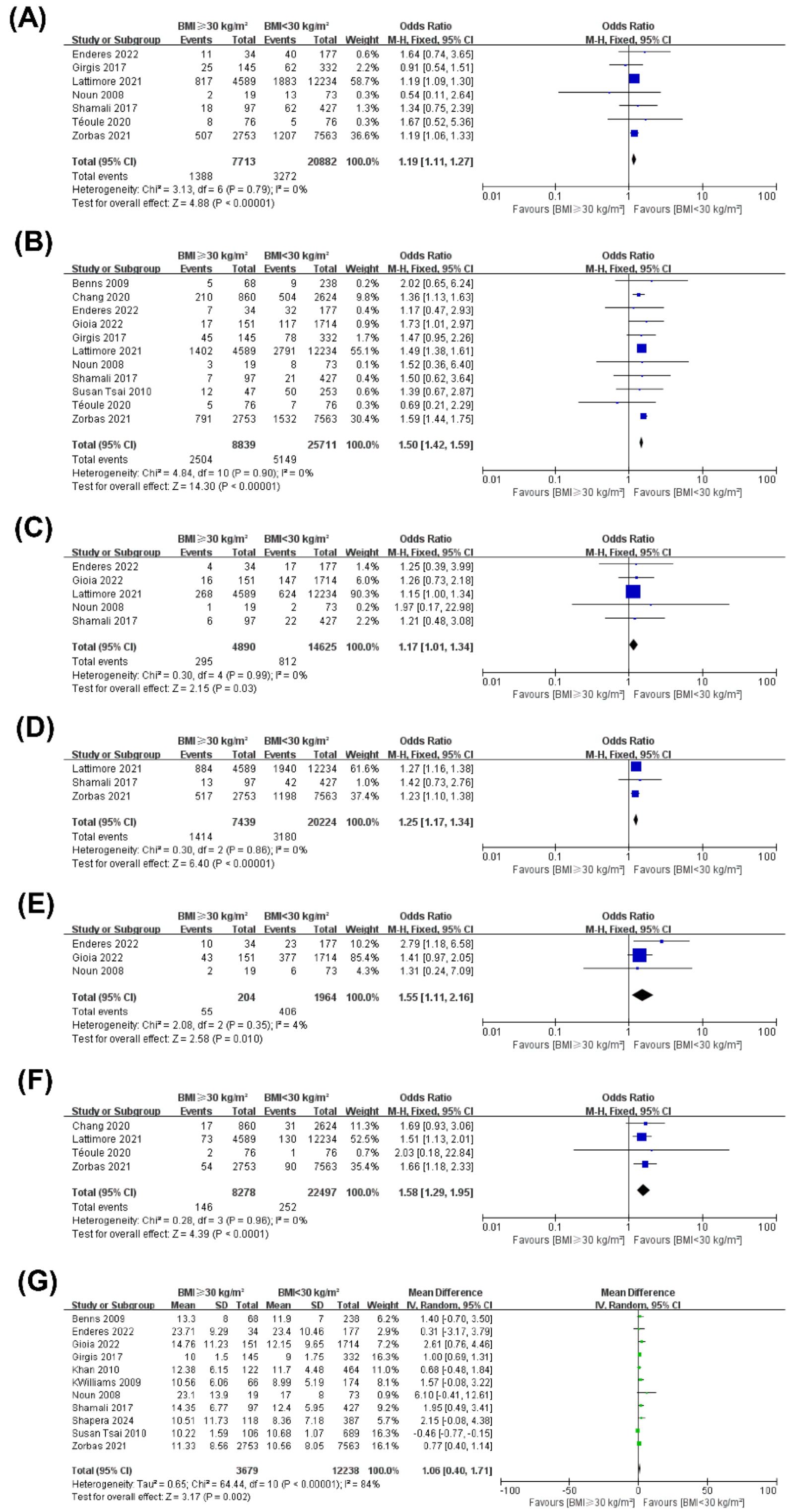

Figure S3. Forest plots showing the association between BMI ≥30 kg/m² and perioperative outcomes not included in the main figures: (A) delayed gastric emptying; (B) wound infection; (C) reoperation rate; (D) re-admission; (E) intra-abdominal abscess; (F) wound dehiscence; (G) postoperative hospital stay (mean difference, days). Effect estimates are presented as ORs or MDs with 95% CIs. Statistical models were applied based on the level of heterogeneity.

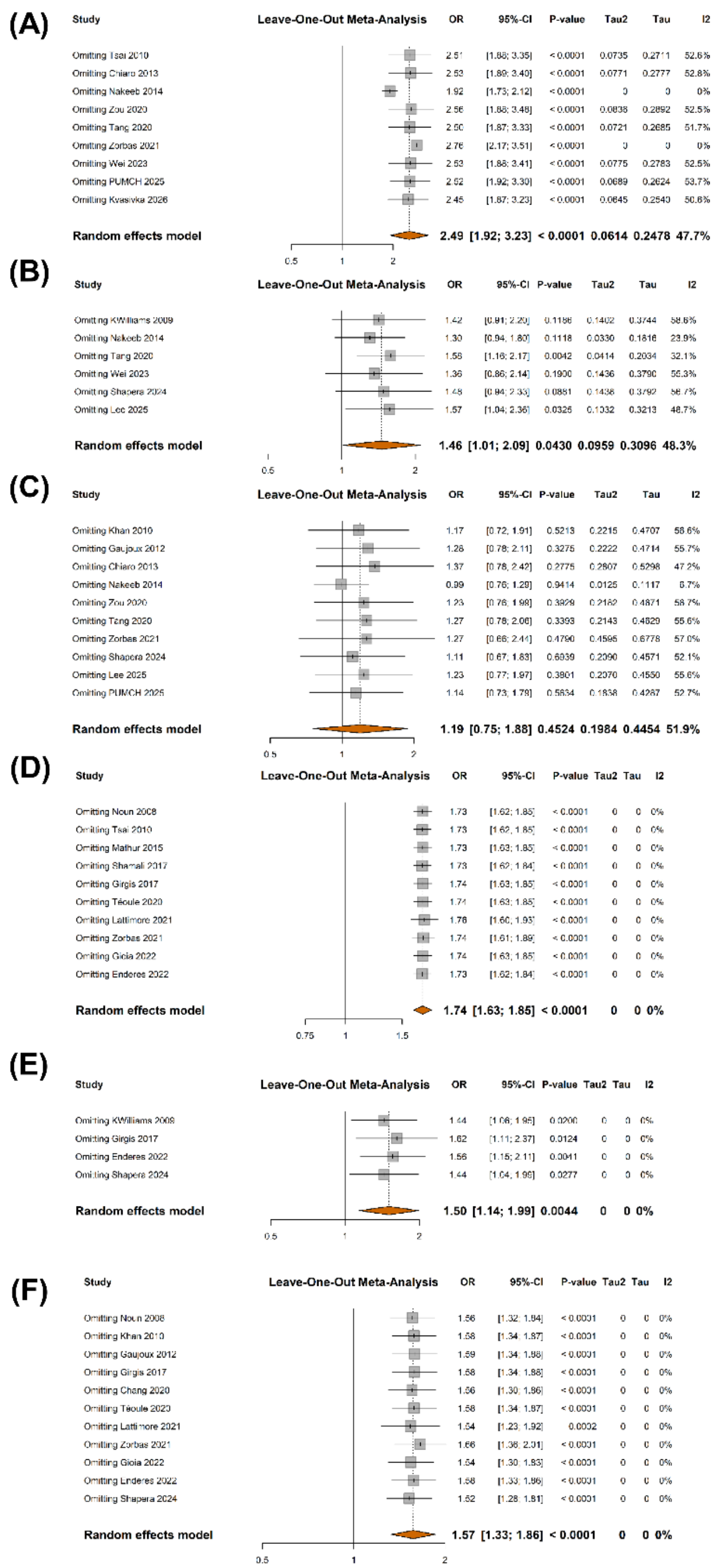

Figure S4. Sensitivity analyses using a leave-one-out approach for primary outcomes in both the BMI ≥25 vs <25 kg/m² (A–C) and BMI ≥30 vs <30 kg/m² (D–F) comparisons, evaluating (A, D) clinically relevant postoperative pancreatic fistula (POPF, grade B/C), (B, E) major complications (Clavien–Dindo grade ≥III), and (C, F) perioperative mortality. Pooled effect estimates were recalculated by sequentially omitting each study to evaluate the robustness of the results; no individual study substantially altered the direction or statistical significance of the pooled estimates.

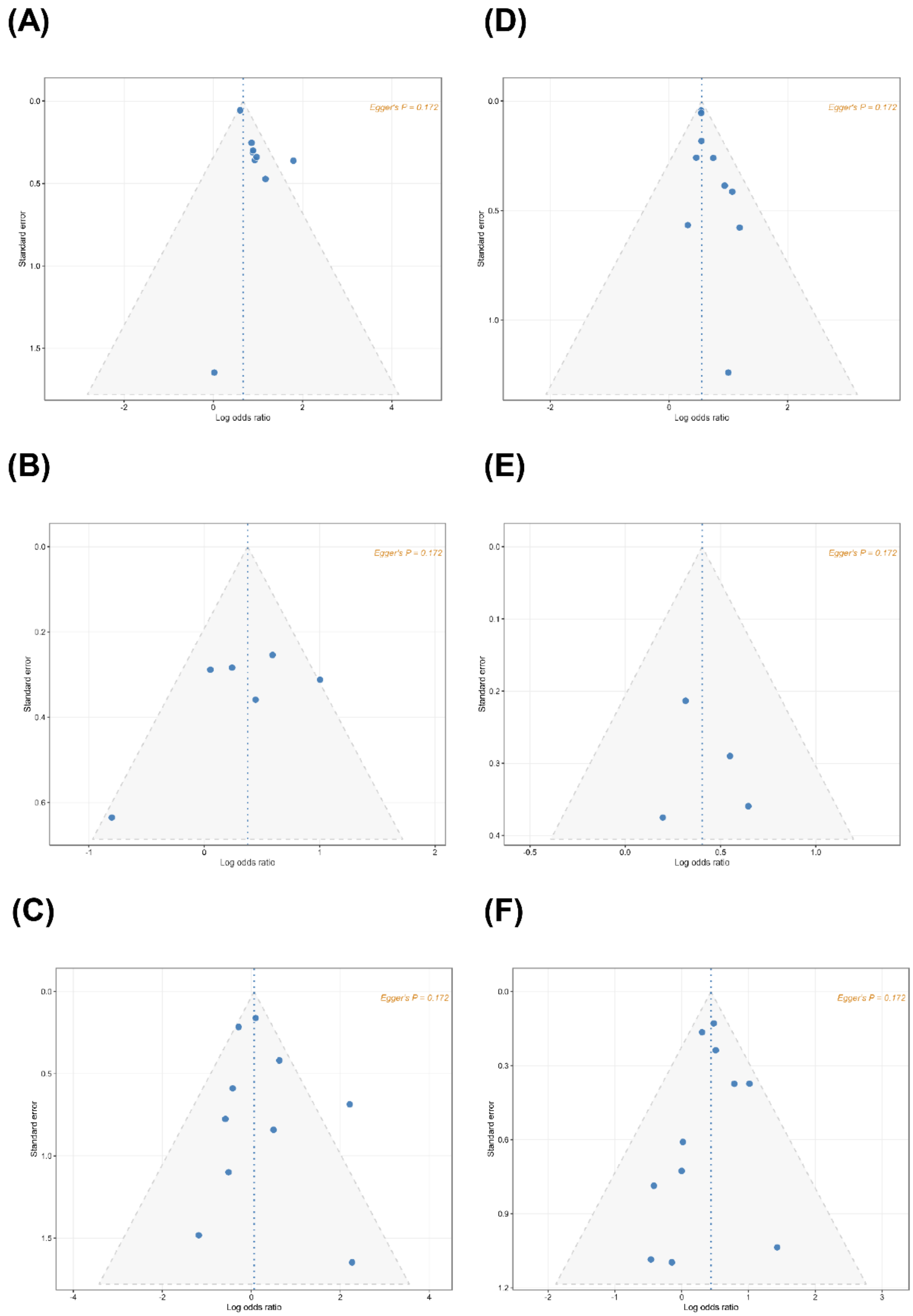

Figure S5. Funnel plots for primary outcomes in the BMI ≥25 vs <25 kg/m² comparison (A–C) and the BMI ≥30 vs <30 kg/m² comparison (D–F), including (A, D) clinically relevant POPF (grade B/C), (B, E) major complications (Clavien–Dindo ≥III), and (C, F) perioperative mortality. Visual inspection revealed no substantial asymmetry; Egger's regression test was non-significant for all primary outcomes (all P >0.10), suggesting no major publication bias.

1.2 Supplementary Tables

Table S1. Detailed search strategy and results for PubMed, Embase, and the Cochrane Library.

Database	Search Strategy	Records
PubMed	(("Obesity"[Mesh] OR "Body Mass Index"[Mesh] OR "Overweight"[Mesh]) OR (obesity[Title/Abstract] OR obese[Title/Abstract] OR overweight[Title/Abstract] OR adiposity[Title/Abstract] OR "BMI"[Title/Abstract] OR "body mass index"[Title/Abstract] OR "body mass"[Title/Abstract] OR "high BMI"[Title/Abstract] OR "elevated BMI"[Title/Abstract])) AND (("Pancreaticoduodenectomy"[Mesh] OR "Pancreatectomy"[Mesh]) OR (pancreaticoduodenectomy[Title/Abstract] OR pancreatoduodenectomy[Title/Abstract] OR whipple[Title/Abstract] OR duodenopancreatectomy[Title/Abstract] OR "pancreatic resection"[Title/Abstract] OR pancreatectomy[Title/Abstract] OR "pancreatic surgery"[Title/Abstract] OR "pancreas resection"[Title/Abstract]))	1431
Embase	('obesity':ab,ti OR 'obese':ab,ti OR 'overweight':ab,ti OR 'adiposity':ab,ti OR 'bmi':ab,ti OR 'body mass index':ab,ti) AND ('pancreaticoduodenectomy':ab,ti OR 'pancreatoduodenectomy':ab,ti OR 'whipple':ab,ti OR 'duodenopancreatectomy':ab,ti OR 'pancreatic resection':ab,ti OR 'pancreatectomy':ab,ti OR 'pancreatic surgery':ab,ti)	3124
Cochrane Library	(MeSH descriptor: [Obesity] explode all trees OR MeSH descriptor: [Body Mass Index] explode all trees OR MeSH descriptor: [Overweight] explode all trees OR (obesity OR obese OR overweight OR adiposity OR BMI OR "body mass index" OR "body mass" OR "high BMI" OR "elevated BMI"):ti,ab,kw) AND (MeSH descriptor: [Pancreaticoduodenectomy] explode all trees OR MeSH descriptor: [Pancreatectomy] explode all trees OR	96

	(pancreaticoduodenectomy OR pancreatoduodenectomy OR whipple OR duodenopancreatectomy OR "pancreatic resection" OR pancreatectomy OR "pancreatic surgery" OR "pancreas resection"):ti,ab,kw)	
Total	Total records identified (Duplicates removed: 1121; Screened: 3530; Included: 25, comprising 24 published studies + 1 institutional cohort)	4651

Notes: All searches were restricted to English-language publications from database inception to March 13, 2026.

Table S2. Detailed quality assessment of included studies using the Newcastle–Ottawa Scale (NOS).

First Author, Year	Selection (0–4)	Comparability (0–2)	Outcome (0–3)	Total Score	Quality
Roger Noun, 2008	3	2	2	7	High
Timothy K. Williams, 2009	4	2	2	8	High
Matthew Benns, 2009	3	2	2	7	High

Susan Tsai, 2010	4	2	3	9	High
Saboor Khan, 2010	4	1	3	8	High
Monica Dandona, 2011	4	2	2	8	High
Sébastien Gaujoux, 2012	3	2	3	8	High
Marco Del Chiaro, 2013	3	3	3	9	High
Ayman El Nakeeb, 2014	4	2	2	8	High
Abhishek Mathur, 2015	3	2	2	7	High
Awad Shamali, 2017	4	1	3	8	High

Mark D. Girgis, 2017	4	1	3	8	High
Tianyu Tang, 2020	3	2	2	7	High
Si-Yi Zou, 2020	4	2	2	8	High
E.H. Chang, 2020	4	2	3	9	High
Patrick Téoule, 2020	4	2	3	9	High
Konstantinos A. Zorbas, 2021	4	2	3	9	High
Courtney M. Lattimore, 2021	4	2	3	9	High
Jana Enderes, 2022	4	1	2	7	High

Anthony Di Gioia, 2022	4	2	2	8	High
Tzu-Hui Wei, 2023	4	2	2	8	High
Emanuel Shapera, 2024	4	2	3	9	High
Boram Lee, 2025	4	2	2	8	High
Oleksandr Kvasivka, 2026	4	1	2	7	High
PUMCH Cohort, 2025	4	2	2	8	High

Notes: Selection domain (maximum 4 points): representativeness and selection of cohorts, ascertainment of exposure. Comparability domain (maximum 2 points): adjustment for age, sex, and surgical factors. Outcome domain (maximum 3 points): assessment of outcome and adequacy of follow-up. Studies scoring ≥7 are considered high quality. Abbreviations: NOS, Newcastle–Ottawa Scale; PUMCH, Peking Union Medical College Hospital.

Table S3. Institutional cohort: clinicopathological and perioperative outcomes after pancreaticoduodenectomy stratified by BMI (≥25 vs <25 kg/m²)

Indicator Category / Specific Item	BMI <25 kg/m²	BMI ≥25 kg/m²	P value	Effect size (BMI <25 vs. ≥25)	95% Confidence Interval (CI)
1. Clinical Baseline Characteristics					
Categorical Variables					
Sex (male), n (%)	49 (53.8%) (n=91)	15 (51.7%) (n=29)	0.842	OR=1.089	0.472 to 2.514
ASA classification (grade III–IV), n (%)	10 (12.3%) (n=81)	3 (12.5%) (n=24)	1.000*	OR=0.986	0.248 to 3.915
Presence of metastasis on admission, n (%)	7 (7.8%) (n=90)	0 (0.0%) (n=29)	0.192*	OR=-	-**
Pancreatic duct dilatation, n (%)	75 (78.9%) (n=91)	20 (66.7%) (n=28)	0.205	OR=1.875	0.703 to 5.004

Smoking history, n (%)	33 (36.3%) (n=91)	8 (27.6%) (n=29)	0.371	OR=1.520	0.605 to 3.815
Alcohol drinking history, n (%)	19 (20.9%) (n=91)	5 (17.2%) (n=29)	0.652	OR=1.285	0.433 to 3.814
Hypertension, n (%)	25 (27.5%) (n=91)	13 (44.8%) (n=29)	0.080	OR=0.466	0.196 to 1.107
Diabetes mellitus, n (%)	29 (31.9%) (n=91)	6 (20.7%) (n=29)	0.249	OR=1.793	0.659 to 4.878
Coronary heart disease, n (%)	12 (13.2%) (n=91)	2 (6.9%) (n=29)	0.358	OR=2.051	0.431 to 9.753
History of pancreatitis, n (%)	5 (5.5%) (n=91)	0 (0.0%) (n=29)	0.334*	OR=-	-**
Hyperlipidemia, n (%)	5 (5.5%) (n=91)	1 (3.4%) (n=29)	1.000*	OR=1.628	0.182 to 14.531
Other comorbidities, n (%)	44 (48.4%) (n=91)	16 (55.2%) (n=29)	0.522	OR=0.761	0.329 to 1.761

Abdominal surgery history, n (%)	21 (23.1%) (n=91)	7 (24.1%) (n=29)	0.906	OR=0.943	0.354 to 2.513
Family history of tumor, n (%)	13 (14.3%) (n=91)	4 (13.8%) (n=29)	0.947	OR=1.042	0.311 to 3.486
Neoadjuvant chemoradiotherapy before surgery, n (%)	2 (2.2%) (n=91)	0 (0.0%) (n=29)	1.000*	OR=-	-**
Surgical approach (laparoscopic vs. open/converted), n (%)	66 (72.5%) (n=91)	27 (93.1%) (n=29)	0.021	OR=0.196	0.043 to 0.884
Intraoperative blood transfusion, n (%)	26 (28.6%) (n=91)	6 (20.7%) (n=29)	0.403	OR=1.533	0.560 to 4.197
Continuous Variables					
Age (years, mean ± SD)	60.08 ± 9.97 (n=91)	57.93 ± 11.71 (n=29)	0.336	MD=2.15	-2.25 to 6.54
BMI (kg/m², mean ± SD)	21.63 ± 2.01 (n=91)	27.00 ± 1.40 (n=29)	<0.001	MD=-5.38	-6.17 to -4.58

Admission systolic blood pressure (mmHg, mean ± SD)	121.73 ± 16.53 (n=91)	127.59 ± 15.65 (n=29)	0.095	MD=-5.86	-12.76 to 1.04
Admission diastolic blood pressure (mmHg, mean ± SD)	74.80 ± 11.04 (n=91)	81.93 ± 10.70 (n=29)	0.003	MD=-7.13	-11.76 to -2.50
2. Pathological Characteristics					
Categorical Variables					
Advanced tumor stage (stage III–IV), n (%)	22 (30.1%) (n=73)	4 (17.4%) (n=23)	0.230	OR=2.049	0.624 to 6.725
R0 resection, n (%)	88 (97.8%) (n=90)	28 (96.6%) (n=29)	0.571*	OR=1.571	0.137 to 17.990
Lymph node metastasis, n (%)	51 (69.9%) (n=73)	13 (56.5%) (n=23)	0.237	OR=1.783	0.680 to 4.677
Continuous Variables					

Tumor size (cm, mean ± SD)	2.79 ± 1.07 (n=70)	3.24 ± 1.35 (n=20)	0.125	MD=-0.45	-1.02 to 0.13
Number of dissected lymph nodes (mean ± SD)	24.81 ± 12.91 (n=73)	23.43 ± 9.22 (n=23)	0.637	MD=1.37	-3.53 to 6.28
3. Perioperative Outcomes					
Continuous Variables					
Operative time (minutes, mean ± SD)	303.57 ± 97.75 (n=91)	317.34 ± 86.56 (n=29)	0.499	MD=-13.77	-54.00 to 26.46
Estimated blood loss (mL, mean ± SD)	512.64 ± 534.15 (n=91)	525.86 ± 415.47 (n=29)	0.903	MD=-13.22	-228.00 to 201.55
Postoperative hospital stay (days, mean ± SD)	12.63 ± 8.00 (n=91)	13.32 ± 9.08 (n=28)	0.698	MD=-0.69	-4.23 to 2.84
4. Postoperative Short-term Outcomes					

Categorical Variables

Postoperative pancreatic fistula, n (%)	1 (1.1%) (n=91)	0 (0.0%) (n=29)	1.000*	OR=-	-**
Surgical site infection, n (%)	1 (1.1%) (n=91)	0 (0.0%) (n=29)	1.000*	OR=-	-**
Postoperative complications, n (%)	16 (17.6%) (n=91)	9 (31.0%) (n=29)	0.120	OR=0.474	0.183 to 1.231
30-day perioperative mortality, n (%)	0 (0.0%) (n=45)	1 (5.9%) (n=17)	0.274*	OR=-	-**
Reoperation, n (%)	2 (2.6%) (n=77)	1 (4.0%) (n=25)	1.000*	OR=0.640	0.056 to 7.373

5. Laboratory Test Results

Blood Routine

White blood cell count ($*10^9$/L, mean ± SD)	6.32 ± 2.51 (n=91)	6.27 ± 1.78 (n=29)	0.914	MD=0.05	-0.94 to 1.05

Neutrophil count (*10^9/L, mean ± SD)	4.30 ± 2.60 (n=91)	3.78 ± 1.45 (n=29)	0.316	MD=0.51	-0.49 to 1.52
Lymphocyte count (*10^9/L, mean ± SD)	1.42 ± 0.51 (n=91)	1.83 ± 0.64 (n=29)	<0.001	MD=-0.41	-0.64 to -0.18
Monocyte count (*10^9/L, mean ± SD)	0.37 ± 0.13 (n=91)	0.35 ± 0.11 (n=29)	0.471	MD=0.02	-0.03 to 0.07
Hemoglobin (g/L, mean ± SD)	126.58 ± 15.19 (n=91)	133.79 ± 14.49 (n=29)	0.026	MD=-7.21	-13.56 to -0.87
Platelet count (*10^{12}/L, mean ± SD)	218.98 ± 67.43 (n=91)	215.14 ± 50.29 (n=29)	0.778	MD=3.84	-23.09 to 30.77
Liver and Kidney Function & Metabolism					
Prealbumin (mg/L, mean ± SD)	197.70 ± 62.61 (n=90)	228.15 ± 56.49 (n=27)	0.025	MD=-30.45	-57.08 to -3.81

Albumin (g/L, mean ± SD)	40.10 ± 3.84 (n=91)	41.83 ± 4.33 (n=29)	0.043	MD=-1.73	-3.40 to -0.06
Total bilirubin (µmol/L, mean ± SD)	53.56 ± 68.15 (n=91)	47.55 ± 62.17 (n=29)	0.674	MD=6.01	-22.19 to 34.21
Direct bilirubin (µmol/L, mean ± SD)	39.12 ± 53.89 (n=91)	33.97 ± 51.15 (n=29)	0.651	MD=5.14	-17.34 to 27.63
ALT (U/L, mean ± SD)	103.96 ± 160.92 (n=91)	96.34 ± 134.28 (n=29)	0.818	MD=7.61	-57.85 to 73.07
AST (U/L, mean ± SD)	73.93 ± 103.79 (n=90)	73.89 ± 92.38 (n=27)	0.998	MD=0.04	-43.99 to 44.08
Uric acid (µmol/L, mean ± SD)	268.48 ± 97.74 (n=90)	320.56 ± 94.18 (n=27)	0.016	MD=-52.08	-94.21 to -9.94
Creatinine (µmol/L, mean ± SD)	62.14 ± 15.56 (n=91)	71.07 ± 17.91 (n=29)	0.011	MD=-8.93	-15.74 to -2.11

Cholinesterase (kU/L, mean ± SD)	5.96 ± 1.95 (n=90)	7.20 ± 1.87 (n=27)	0.004	MD=-1.25	-2.09 to -0.41
hsCRP (mg/L, mean ± SD)	12.52 ± 22.57 (n=81)	15.80 ± 39.59 (n=28)	0.592	MD=-3.28	-15.39 to 8.83
Fasting blood glucose (mmol/L, mean ± SD)	7.14 ± 2.69 (n=91)	6.81 ± 2.01 (n=29)	0.544	MD=0.33	-0.74 to 1.41
Triglyceride (mmol/L, mean ± SD)	1.54 ± 0.86 (n=83)	1.92 ± 1.08 (n=29)	0.061	MD=-0.38	-0.77 to 0.02
LDL-C (mmol/L, mean ± SD)	2.87 ± 1.24 (n=83)	2.89 ± 1.35 (n=29)	0.947	MD=-0.02	-0.56 to 0.53
HDL-C (mmol/L, mean ± SD)	5.72 ± 42.65 (n=79)	0.86 ± 0.22 (n=27)	0.557	MD=4.85	-11.48 to 21.18
Free fatty acid (µmol/L, mean ± SD)	595.01 ± 307.32 (n=82)	576.54 ± 381.21 (n=29)	0.795	MD=18.47	-121.94 to 158.88
Coagulation Function					

Fibrinogen (g/L, mean ± SD)	3.55 ± 0.96 (n=90)	3.38 ± 1.00 (n=29)	0.412	MD=0.17	-0.24 to 0.58
D-Dimer (mg/L, mean ± SD)	1.09 ± 1.61 (n=89)	1.04 ± 1.05 (n=29)	0.865	MD=0.05	-0.58 to 0.69
PT (seconds, mean ± SD)	12.28 ± 1.15 (n=90)	11.93 ± 1.05 (n=29)	0.152	MD=0.35	-0.13 to 0.82
APTT (seconds, mean ± SD)	28.22 ± 7.41 (n=90)	26.53 ± 2.53 (n=29)	0.233	MD=1.69	-1.10 to 4.47
INR (mean ± SD)	1.04 ± 0.10 (n=90)	1.01 ± 0.09 (n=29)	0.134	MD=0.03	-0.01 to 0.07
Tumor Markers					
AFP (ng/mL, mean ± SD)	3.30 ± 1.94 (n=60)	2.37 ± 1.08 (n=18)	0.057	MD=0.93	-0.03 to 1.88

CEA (ng/mL, mean ± SD)	4.99 ± 5.81 (n=84)	3.81 ± 4.26 (n=26)	0.342	MD=1.18	-1.27 to 3.62
CA199 (U/mL, mean ± SD)	356.58 ± 1022.30 (n=83)	457.62 ± 1434.11 (n=26)	0.692	MD=-101.04	-605.38 to 403.30
CA242 (U/mL, mean ± SD)	42.98 ± 72.66 (n=82)	21.97 ± 24.54 (n=24)	0.168	MD=21.01	-8.97 to 50.99
CA125 (U/mL, mean ± SD)	54.04 ± 91.14 (n=71)	68.28 ± 163.37 (n=22)	0.604	MD=-14.24	-68.53 to 40.06
6. Long-term Survival Outcomes					
Overall survival (HR)	Median 36.00 months (n=91)	Median 40.00 months (n=29)	0.698	HR=1.14	0.5676 to 2.297

Notes: Continuous variables: mean ± SD (independent samples t-test); categorical variables: n (%) (Pearson's chi-square test). *Fisher's exact test (two-tailed P-values) for expected cell count <5 (sparse data). Effect sizes: OR (categorical variables), MD (continuous variables), HR (overall survival, Cox proportional hazards model). All effect sizes compare BMI ≥25 kg/m² (exposure group) vs. BMI <25 kg/m² (reference group). **OR/95% CI not calculable for variables with zero events in one group (pancreatic fistula, surgical site infection, history of pancreatitis, neoadjuvant chemoradiotherapy). n = valid cases; sample size differences due to missing data. Abbreviations: PDAC = pancreatic ductal adenocarcinoma; BMI = body mass index; SD = standard deviation; CI = confidence interval; ASA = American Society of

Anesthesiologists; OR = odds ratio; MD = mean difference; HR = Hazard Ratio; LDL-C = low-density lipoprotein cholesterol; HDL-C = high-density lipoprotein cholesterol; AFP = alpha-fetoprotein; CEA = carcinoembryonic antigen; PT = prothrombin time; APTT = activated partial thromboplastin time; INR = international normalized ratio.

Table S4. Exploratory pairwise comparisons across three BMI categories.

Indicator Category / Specific Item	25–30 vs. <25 (Overweight vs. Normal)OR/MD/HR [95% CI]; P (No. of studies)	>30 vs. <25 (Obese vs. Normal)OR/MD/HR [95% CI]; P (No. of studies)	>30 vs. 25–30 (Obese vs. Overweight)OR/MD/HR [95% CI]; P (No. of studies)
**1. Clinical Baseline Characteristics**			
Age (years)	MD=0.36 [-1.03, 1.75]; 0.61 (n=7)	MD=-1.20 [-3.23, 0.83]; 0.25 (n=6)	MD=-2.34 [-2.85, -1.83]; <0.00001 (n=6)
Sex (male)	OR=1.61 [1.49, 1.74]; <0.00001 (n=8)	OR=1.08 [0.99, 1.18]; 0.08 (n=7)	OR=0.66 [0.61, 0.73]; <0.00001 (n=7)
Preoperative weight loss	OR=0.73 [0.56, 0.94]; 0.02 (n=4)	OR=0.65 [0.42, 1.02]; 0.06 (n=4)	OR=0.77 [0.68, 0.89]; 0.0002 (n=4)
ASA class III–IV	OR=1.02 [0.92, 1.12]; 0.71 (n=5)	OR=1.55 [1.39, 1.74]; <0.00001 (n=4)	OR=1.51 [1.34, 1.70]; <0.00001 (n=3)
**2. Tumor Pathological Characteristics**			
Tumor size (cm)	MD=-0.01 [-0.04, 0.03]; 0.68 (n=5)	MD=0.03 [-0.03, 0.09]; 0.29 (n=4)	MD=0.05 [-0.10, 0.19]; 0.54 (n=4)
R0 resection	OR=0.95 [0.83, 1.32]; 0.69 (n=4)	OR=0.96 [0.56, 1.65]; 0.80 (n=3)	OR=1.05 [0.85, 1.29]; 0.80 (n=3)

Lymph node metastasis	OR=0.90 [0.73, 1.11]; 0.32 (n=5)	OR=0.93 [0.71, 1.22]; 0.60 (n=4)	OR=1.01 [0.76, 1.32]; 0.97 (n=4)
**3. Perioperative Operative Outcomes**			
Operative time (minutes)	MD=13.31 [8.36, 18.26]; <0.00001 (n=6)	MD=31.12 [25.53, 36.72]; <0.00001 (n=5)	MD=13.44 [-0.58, 27.46]; 0.06 (n=5)
Estimated blood loss (mL)	MD=103.40 [89.17, 117.63]; <0.00001 (n=6)	MD=207.48 [122.07, 292.89]; <0.0001 (n=5)	MD=100.98 [19.96, 181.99]; 0.01 (n=5)
**4. Postoperative Short-term Outcomes**			
Perioperative mortality	OR=1.01 [0.73, 1.39]; 0.95 (n=4)	OR=1.41 [1.02, 1.95]; 0.04 (n=4)	OR=1.45 [1.04, 2.03]; 0.03 (n=4)
Postoperative complications	OR=1.23 [1.13, 1.35]; <0.00001 (n=4)	OR=1.42 [1.29, 1.57]; <0.00001 (n=3)	OR=1.16 [1.05, 1.28]; 0.004 (n=3)
Postoperative hospital stay (days)	MD=-0.17 [-1.02, 0.68]; 0.69 (n=6)	MD=0.69 [-0.50, 1.88]; 0.26 (n=5)	MD=0.34 [0.09, 0.59]; 0.007 (n=5)
**5. Long-term Survival Outcomes**			
Overall survival (OS)	HR=0.98 [0.74, 1.30]; 0.88 (n=4)	HR=0.93 [0.70, 1.24]; 0.62 (n=3)	HR=0.93 [0.78, 1.10]; 0.41 (n=3)

Notes: Only comparisons supported by at least three studies are shown. These analyses are exploratory. OR, odds ratio; MD, mean difference; HR, hazard ratio; CI, confidence interval; n, number of included studies.

Table S5. Subgroup analyses of clinically relevant postoperative pancreatic fistula.

BMI comparison	Subgroup factor	Category	k	OR (95% CI)	I²	P for interaction
**BMI ≥25 vs <25**						
	ISGPS/ISGPF definition	Unclear	1	2.53 [1.25, 5.12]	—	**0.018**
		No	2	1.84 [1.65, 2.05]	0.0%	
		Yes	6	2.91 [2.14, 3.95]	11.0%	
	Surgical approach	Open-only	4	2.94 [1.95, 4.43]	42.6%	**0.027**
		Mixed/MIPD-inclusive	4	2.60 [1.75, 3.86]	0.0%	
		Unclear	1	1.82 [1.63, 2.04]	—	
	Formal surgical comparison	Open-only vs Mixed/MIPD-inclusive	8	—	—	0.671
	Geographic region	North America	2	1.84 [1.65, 2.05]	0.0%	**0.003**
		Europe	2	2.64 [1.58, 4.39]	0.0%	
		Asia	4	2.43 [1.75, 3.37]	0.0%	

BMI comparison	Subgroup factor	Category	k	OR (95% CI)	I²	P for interaction
		Other	1	6.02 [2.96, 12.24]	—	
	Publication year	<2015	3	3.28 [1.86, 5.79]	53.3%	0.062
		≥2015	6	1.89 [1.70, 2.10]	0.0%	
**BMI ≥30 vs <30**						
	ISGPS/ISGPF definition	Yes	7	1.74 [1.60, 1.88]	0.0%	0.598
		Unclear	1	2.55 [1.20, 5.44]	—	
		No	2	1.72 [1.55, 1.91]	0.0%	
	Surgical approach	Open-only	6	2.06 [1.60, 2.65]	0.0%	0.362
		Mixed/MIPD-inclusive	1	1.58 [0.95, 2.63]	—	
		Unclear	3	1.72 [1.61, 1.84]	0.0%	
	Formal surgical comparison	Open-only vs Mixed/MIPD-inclusive	7	—	—	0.359
	Geographic region	North America	5	1.72 [1.61, 1.84]	0.0%	0.419

BMI comparison	Subgroup factor	Category	k	OR (95% CI)	I²	P for interaction
		Europe	4	1.90 [1.46, 2.49]	0.0%	
		Other	1	3.29 [1.06, 10.19]	—	
	Publication year	<2015	2	2.76 [1.47, 5.18]	0.0%	0.146
		≥2015	8	1.73 [1.62, 1.84]	0.0%	

Notes: Random-effects subgroup estimates are shown. The interaction P value is displayed in the first row of each subgroup factor. Single-study categories are presented descriptively and have no pooled I² estimate. MIPD, minimally invasive pancreaticoduodenectomy.

Table S6. Subgroup analyses of primary outcomes by surgical approach.

BMI comparison	Outcome	Open-only OR (95% CI), k	Mixed/MIPD-inclusive OR (95% CI), k	P for interaction
**BMI ≥25 vs <25**				
	Overall complications	1.71 [1.20, 2.44] (k=3)	2.11 [0.81, 5.48] (k=1)	0.686
	Major complications	2.12 [1.23, 3.66] (k=2)	1.25 [0.70, 2.23] (k=3)	0.193
	Clinically relevant POPF	2.94 [1.95, 4.43] (k=4)	2.60 [1.75, 3.86] (k=4)	0.671
	Delayed gastric emptying	1.59 [1.16, 2.18] (k=3)	1.88 [0.78, 4.56] (k=3)	0.726
	Postoperative hemorrhage	1.38 [0.87, 2.21] (k=3)	2.21 [1.16, 4.20] (k=2)	0.252
	Perioperative mortality	1.32 [0.51, 3.42] (k=5)	1.50 [0.50, 4.50] (k=3)	0.862
**BMI ≥30 vs <30**				
	Overall complications	1.48 [1.20, 1.82] (k=6)	Not available	Not estimable
	Major complications	1.54 [0.93, 2.56] (k=2)	1.49 [1.06, 2.09] (k=2)	0.918

BMI comparison	Outcome	Open-only OR (95% CI), k	Mixed/MIPD-inclusive OR (95% CI), k	P for interaction
	Clinically relevant POPF	2.06 [1.60, 2.65] (k=6)	1.58 [0.95, 2.63] (k=1)	0.359
	Delayed gastric emptying	1.33 [0.85, 2.08] (k=3)	0.91 [0.54, 1.51] (k=1)	0.273
	Postoperative hemorrhage	1.29 [0.74, 2.24] (k=3)	Not available	Not estimable
	Perioperative mortality	1.66 [0.93, 2.97] (k=5)	1.88 [0.73, 4.87] (k=2)	0.827

Notes: Cohorts including MIPD comprised studies involving laparoscopic or robotic pancreaticoduodenectomy, either alone or in combination with open procedures. MIPD, minimally invasive pancreaticoduodenectomy. Studies with unclear surgical approach were excluded from the formal interaction test. “Not estimable” indicates that no mixed/minimally invasive-inclusive cohort contributed to the outcome. MIPD, minimally invasive pancreaticoduodenectomy.

Table S7. Sensitivity analyses excluding the investigator-contributed PUMCH cohort.

Outcome	PUMCH included	k	Effect measure	Pooled effect (95% CI)	I²
Overall complications	Yes	5	OR	1.53 [1.20, 1.96]	46.9%
	No	4	OR	1.51 [1.16, 1.97]	54.7%
Clinically relevant POPF	Yes	9	OR	2.49 [1.92, 3.23]	47.7%
	No	8	OR	2.52 [1.92, 3.30]	53.7%
Delayed gastric emptying	Yes	7	OR	1.52 [1.22, 1.89]	30.0%
	No	6	OR	1.44 [1.23, 1.68]	11.5%
Postoperative hemorrhage	Yes	5	OR	1.63 [1.11, 2.38]	0.0%

Outcome	PUMCH included	k	Effect measure	Pooled effect (95% CI)	I²
Perioperative mortality	No	4	OR	1.63 [1.11, 2.39]	0.0%
	Yes	10	OR	1.19 [0.75, 1.88]	51.9%
	No	9	OR	1.14 [0.73, 1.79]	52.7%
Reoperation	Yes	3	OR	1.94 [0.99, 3.80]	17.8%
	No	2	OR	2.05 [0.80, 5.25]	58.5%
Wound infection	Yes	6	OR	1.51 [1.38, 1.66]	0.0%
	No	5	OR	1.51 [1.38, 1.66]	0.0%
Operative time (minutes)	Yes	12	MD	18.78 [14.18, 23.38]	42.6%
	No	11	MD	18.95 [14.16, 23.75]	47.8%
Estimated blood loss (mL)	Yes	12	MD	108.17 [48.74, 167.60]	93.6%
	No	11	MD	113.26 [52.37, 174.15]	94.1%
Postoperative hospital stay (days)	Yes	11	MD	0.96 [-0.45, 2.38]	98.9%
	No	10	MD	0.98 [-0.48, 2.45]	99.0%

Notes: The PUMCH cohort contributed only to the BMI ≥25 vs <25 kg/m² comparison. OR, odds ratio; MD, mean difference; PUMCH, Peking Union Medical College Hospital.
